# A Spectral-Index-Aligned Transfer-Learning Approach for Automated Plantation Segmentation in High-Resolution Remote-Sensing Imagery

**DOI:** 10.3390/plants15172688

**Published:** 2026-09-01

**Authors:** Ronghui Liu, Tao Liu, Yonglin Xia, Peng Wang, Mingfang He

**Affiliations:** Institute of Applied Artificial Intelligence, Central South University of Forestry and Technology, Changsha 410004, China; 20245166@csuft.edu.cn (R.L.); liutao20051020@gmail.com (T.L.); t20162306@csuft.edu.cn (M.H.)

**Keywords:** plantation segmentation, transfer learning, high-resolution remote sensing, vegetation indices, boundary-aware segmentation, spectral-index alignment

## Abstract

Plantations play an essential role in regional ecological security and the sustainable use of forest resources, making accurate knowledge of their spatial distribution and management boundaries critical for forest inventories, afforestation assessment, carbon-accounting support, and stand-level management. To address blurred stand boundaries, complex background interference, and regional spectral variation, we propose a plantation segmentation framework termed the Kernel-Guided Forest Segmentation Network (KGF-Net). The method combines a spectral-index aligned fusion module (SIAF), a structure-aware boundary enhancement module (SABE), a cross-domain discriminative geometry-constrained coupling module (CDGC), and an Adam-compatible forest-aware responsive spectral control strategy (FReSCO). In the source-domain stage, KGF-Net is pretrained on the North American subset using aligned RGB, NDVI, and EVI inputs, enabling the model to learn vegetation-sensitive spectral and structural representations. For Asia, Europe, Africa, and public FAIS target datasets, no target-domain vegetation-index layers are used; instead, the source-domain weights are transferred and the model is fine-tuned separately using target-domain RGB images and plantation masks. Thus, the cross-region experiments evaluate source-to-target transfer learning with RGB-based target adaptation rather than zero-shot generalization or independent multimodal training in every region. Experiments show that KGF-Net achieves competitive segmentation and boundary-delineation performance while maintaining a compact computational profile. The resulting maps provide spatially explicit information on plantation extent, patch configuration, and management boundaries, supporting regional plantation monitoring and forest-management applications.

## 1. Introduction

Plantation forests are an important component of global forest resources and are established for timber production, ecological restoration, shelterbelt construction, and other management objectives. Spatially explicit information on plantation extent and stand boundaries is required for forest-resource inventories, afforestation-performance assessment, management-unit delineation, disturbance monitoring, and carbon-accounting support. Unlike general forest-cover mapping, plantation mapping must identify managed tree stands and distinguish them from natural forests, orchards, agroforestry systems, shrubs, and other woody or semi-woody vegetation. This distinction depends not only on canopy greenness, but also on planting regularity, crown-spacing patterns, stand homogeneity, and visible management boundaries.

With the increasing availability of high-resolution satellite and aerial imagery, large-scale and fine-grained plantation mapping has become more feasible. However, accurately delineating plantation boundaries remains challenging because plantations may exhibit fragmented edges, overlapping crowns, variable canopy density, spectral differences among regions and acquisition periods, and strong interference from surrounding non-forest backgrounds. These errors can propagate into estimates of plantation area, patch fragmentation, and management-boundary configuration. Therefore, more precise and automated segmentation methods are needed to provide reliable spatial data for plantation monitoring and forest management.

Earlier forest-mapping studies commonly combined spectral, textural, and geometric descriptors with conventional classifiers. For example, knowledge-based rules and random forests were applied to PALSAR-2, Sentinel-2, and Landsat data for eucalyptus plantation mapping [1], and Sentinel-2 data were used with random forests for land-use, land-use-change, and forestry classification [2]. Such methods remain useful when class boundaries and spectral distributions are stable, but shallow descriptors are less effective in scenes with fragmented stands, mixed vegetation, and strong within-class variation.

With the rapid development of deep learning, plantation segmentation and forest mapping have attracted increasing attention. Several studies have investigated plantation delineation from satellite images, especially at medium spatial resolutions. Deep segmentation models have been applied to Sentinel-2 imagery for eucalyptus plantation mapping [3,4]. Annual Landsat reflectance composites have been used for regional vegetation and forest-type mapping [5], and Sentinel-2-based semantic segmentation has been explored for forest classification [6]. Although these methods improve the automation and representation ability of plantation mapping, they are still constrained by moderate spatial resolution, binary classification settings, or insufficient cross-region validation. These limitations reduce their ability to preserve small plantation patches and sharp boundaries.

At higher spatial resolutions, deep-learning-based methods provide stronger potential for fine-scale forest characterization. For instance, GF-1 optical imagery has been combined with deep learning to map forest types in southern China [7]. However, many existing methods still rely mainly on optical images alone. Their limited use of auxiliary information, such as vegetation indices or multisource data, restricts their ability to distinguish plantations from visually similar vegetation types. Although deep-learning-based approaches have made clear progress in plantation-cover identification and spatial-distribution mapping, several limitations remain when they are applied to fine-grained plantation segmentation in high-resolution remote-sensing imagery.

**Figure 1:**
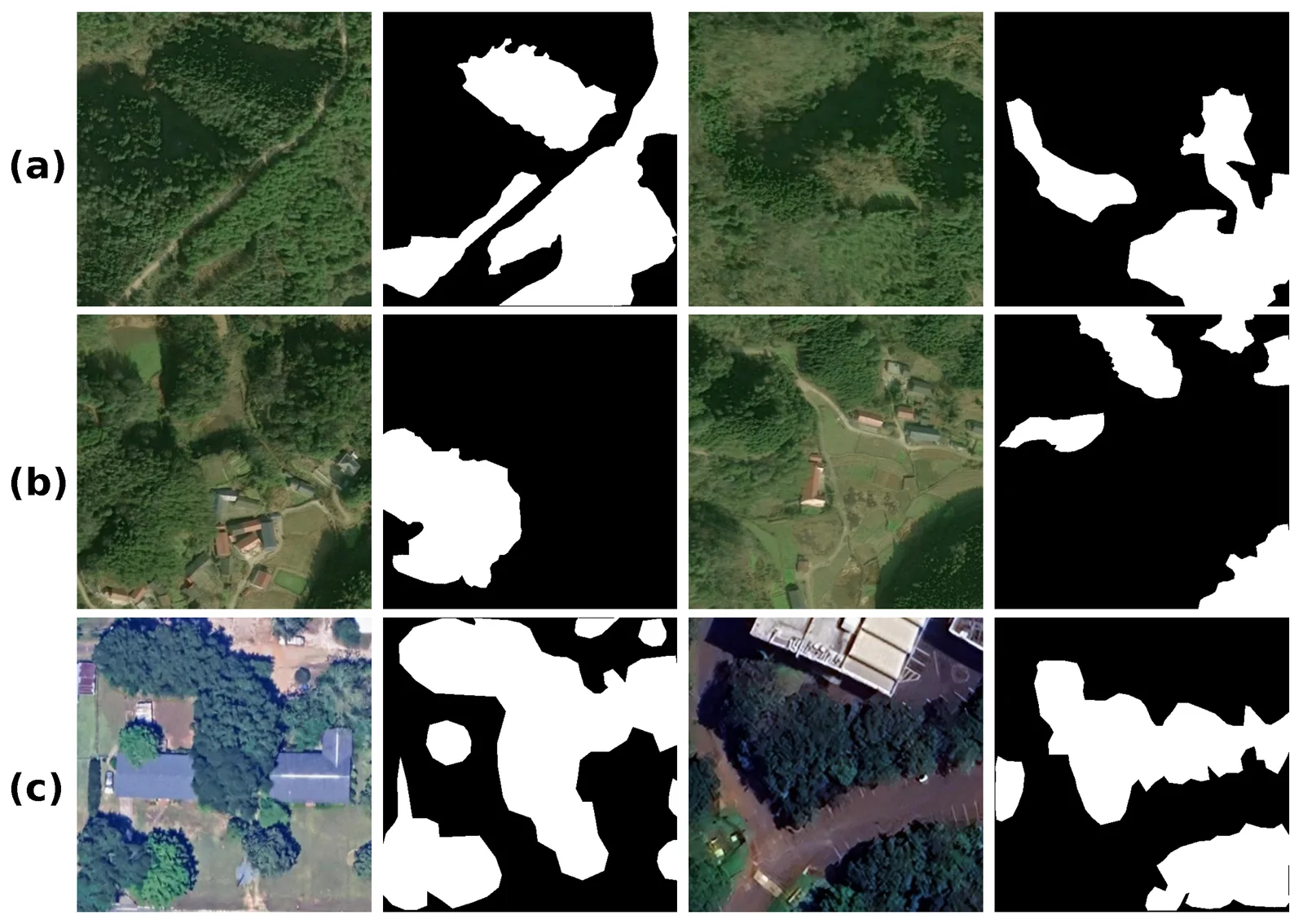
Main challenges in high-resolution plantation segmentation. (**a**) Blurred and fragmented plantation boundaries. (**b**) Background interference from non-plantation objects with similar appearance. (**c**) Spectral and illumination variations across regions. Each row shows representative remote-sensing imagery and the corresponding binary plantation mask.

First, existing methods usually rely on optical-only inputs, and the amount of exploitable information is limited. Single-modal networks may struggle to distinguish plantations from natural forests, shrubs, and grasslands when their visual appearance is similar. Second, plantation boundaries are often irregular and fragmented. As shown in Figure 1, plantations may have unclear edges near natural forests, grassland, or mixed vegetation, leading to jagged boundaries, broken contours, and incomplete segmentation results. Third, complex background interference is common. Houses, farmland, bare soil, roads, and built-up areas often appear close to plantation regions and may share similar gray-level or texture patterns, which increases the risk of false segmentation. Fourth, spectral imbalance across sensors, regions, and acquisition times causes plantations to appear differently in different images, weakening the cross-region generalization ability of segmentation models.

To address the limitation of single-modal optical inputs, multimodal remote-sensing methods have been increasingly studied. Recent studies have jointly modeled LiDAR, SAR, and optical data for forest-height mapping, developed unified multimodal semantic segmentation frameworks for remote sensing, and introduced multilevel feature fusion to align multimodal representations across encoder–decoder hierarchies [8,9,10]. However, most of these methods focus on forest height inversion or general land-cover segmentation. They usually emphasize global scene semantics or multisensor structural information, but lack dedicated spectral characterization for distinguishing plantations from fine-grained vegetation categories, such as shrubs, grasslands, and natural forests. In addition, multimodal fusion is often implemented by simple feature stacking or scale-level fusion, without sufficient pixel-level alignment or coherent cross-modal interaction. To alleviate this issue, this study introduces NDVI and EVI as complementary modalities during North American source-domain pretraining. The vegetation-sensitive representations learned in this stage are subsequently transferred to RGB-only target-domain fine-tuning in Asia, Europe, Africa, and FAIS.

To address blurred and fragmented boundaries, many recent studies have introduced explicit boundary modeling and prediction refinement. Edge-detail enhancement networks, boundary-enhanced UNetFormer variants, and boundary-aware attention fusion networks have been proposed to strengthen contour supervision and boundary responses in remote-sensing semantic segmentation [11,12,13]. Nevertheless, these methods are often designed for objects with relatively regular boundaries, such as buildings and roads. Plantation scenes are more complex because forest edges can be fragmented, shelterbelts can be slender, and canopies may overlap with surrounding vegetation. Under canopy overlap, shadow interference, and texture perturbation, existing boundary-aware methods may still produce over-smoothed or locally missing boundaries. Therefore, a structure-aware boundary modeling strategy is required to preserve plantation-edge continuity and topological consistency.

To reduce background interference, attention mechanisms and feature-reweighting methods have also been widely explored in remote-sensing segmentation. Channel attention, dual-attention fusion, and Transformer–CNN fusion strategies have been used to enhance target responses and balance global and local features [14,15,16]. However, conventional attention modules may still be unstable under multi-scale variation and abrupt background changes. In highly mixed scenes, background regions such as bare soil, roads, buildings, and sparse vegetation may still activate semantic responses similar to plantations. Therefore, a more discriminative feature-coupling strategy is needed to suppress false activations from confusing backgrounds while preserving true plantation canopy responses.

Cross-region training also requires attention to class imbalance and domain-dependent appearance. Semantic-aware region losses and uncertainty-driven example mining have been used to improve regional consistency and remote-sensing domain adaptation [17,18]. These approaches operate mainly through the loss or sample-selection mechanism. In this study, we examine a complementary training-time gradient modulation strategy designed for the spectral and structural variation observed in plantation imagery.

From a plant-science and forestry perspective, the segmentation cues used in this study have direct interpretations. NDVI and EVI provide complementary information on canopy greenness, density, and vegetation condition, whereas the regular spatial arrangement of crowns and the continuity of stand edges reflect plantation establishment and silvicultural management. At the same time, natural forests, orchards, cropland, bare soil, roads, and settlements may share individual spectral or textural characteristics with plantations. Reliable plantation mapping, therefore, requires the joint use of vegetation-related spectral information, stand-structure patterns, boundary continuity, and contextual background discrimination.

To address these problems, this paper proposes a new high-resolution plantation remote-sensing segmentation model, termed the Kernel-Guided Forest Segmentation Network (KGF-Net). The methodological novelty of KGF-Net does not lie in merely combining several generic attention or graph modules. Instead, it lies in the coordinated design of four plantation-oriented mechanisms and their integration into a multimodal-source-to-RGB-target transfer-learning framework. Specifically, SIAF performs same-scale alignment between optical features and vegetation-index features during source-domain pretraining; SABE introduces a hybrid static–dynamic relational graph for plantation-boundary continuity; CDGC jointly couples channel, multi-scale spatial, and global contextual responses to suppress plantation-like backgrounds; and FReSCO modulates Adam gradients according to boundary, texture, spectral, and scale cues without adding inference-time computation. These components address complementary failure modes and are transferred together from the North American multimodal source domain to RGB-only target regions. The main contributions are as follows:

1. We propose a spectral-index-aligned fusion module, termed SIAF, which aligns high-resolution optical imagery with NDVI and EVI during source-domain pretraining. By combining crown-level spatial detail with vegetation-related spectral responses, SIAF learns transferable plantation representations that can subsequently be adapted to RGB-only target regions.
2. We propose a structure-aware boundary enhancement module, termed SABE, which combines static grid-based adjacency with dynamic semantic adjacency to construct a hybrid relational graph. SABE enhances the continuity of plantation edges, narrow strips, and management boundaries under canopy overlap, fragmented stands, and local texture perturbations.
3. We propose a cross-domain discriminative geometry-constrained coupling module, termed CDGC, which jointly models channel–spatial response relationships across multiple scales. This design suppresses interference from bare soil, roads, buildings, cropland, and other confusing backgrounds while preserving canopy responses associated with managed plantation stands.
4. We introduce a forest-aware responsive spectral control strategy, termed FReSCO. Rather than serving as an independent optimizer, FReSCO acts as a gradient-modulation mechanism implemented on top of Adam. Without modifying the network topology, it regulates difficulty-adaptive gradient updates and improves robustness under cross-regional spectral variation.
5. We establish a high-resolution remote-sensing plantation segmentation dataset covering North America, Asia, Europe, and Africa. Using North American RGB–NDVI–EVI pretraining followed by separate RGB-only fine-tuning in each target region, the experiments demonstrate the feasibility of KGF-Net for extracting plantation extent, patch configuration, and stand boundaries at fine spatial scales.

## 2. Materials and Methods

### 2.1. Data Collection

The plantation remote-sensing dataset constructed in this study covers representative plantation regions across four continents, namely North America, Asia, Europe, and Africa. All regional subsets contain high-resolution optical imagery and pixel-level plantation masks. Vegetation-index layers were generated and used only for the North American source-domain subset, which served as the multimodal pretraining domain. The Asia, Europe, and Africa subsets were used as RGB target domains for separate fine-tuning and evaluation.

The North American subset covers Stewart County, Georgia, USA, and the surrounding area, approximately $31.85^{\circ }$–$32.25^{\circ }$ N and $84.65^{\circ }$–$85.05^{\circ }$ W. It was derived from 2021 USDA National Agriculture Imagery Program (NAIP) aerial imagery with an approximate spatial resolution of 0.6 m and red, green, blue, and near-infrared bands. The dominant plantation types include pine plantations and managed conifer–broadleaf stands.

The Asia, Europe, and Africa subsets were derived primarily from high-resolution Google satellite basemap imagery accessed in October 2025 using Omap 9.8 at map level 18. The reported date represents the basemap access and retrieval time rather than the acquisition date of the underlying satellite imagery. Since Google satellite basemap products are distributed as web-based mosaics, the original sensor identity, native spatial resolution, spectral-band metadata, and exact acquisition timestamps of individual tiles were not consistently available. Therefore, these unavailable metadata were not inferred.

The exported map-level-18 imagery corresponds to approximate ground sampling intervals of 0.53 m/pixel for Asia, 0.58 m/pixel for Europe, and 0.59 m/pixel for Africa. These values represent the exported image scale used in this study rather than the native resolution of the original satellite sensors. The Asia subset is located in the Qinglang area of Yuanling County, Huaihua City, Hunan Province, China, approximately $28.25^{\circ }$–$28.65^{\circ }$ N and $110.15^{\circ }$–$110.65^{\circ }$ E. The imagery used for this subset corresponds approximately to the 2020–2022 period and mainly covers eucalyptus, Chinese fir, and mixed conifer–broadleaf plantations. The Europe subset covers East Hampshire, UK, approximately $50.85^{\circ }$–$51.15^{\circ }$ N and $0.75^{\circ }$–$1.15^{\circ }$ W. The imagery corresponds approximately to the 2019–2022 period and mainly contains conifer and broadleaf plantations. The Africa subset covers the Mecha area of Ethiopia, approximately $11.20^{\circ }$–$11.65^{\circ }$ N and $37.00^{\circ }$–$37.50^{\circ }$ E. The imagery corresponds approximately to the 2020–2022 period and mainly includes eucalyptus plantations and managed tree stands in agro-pastoral transition landscapes.

For these Google-basemap subsets, the reported spatial scale refers to the approximate ground sampling interval of the exported map-level-18 imagery rather than the native spatial resolution of the original satellite sensors. It was estimated from the standard Web-Mercator ground-resolution relationship,

(1) $$r\left(\phi ,z\right)=\frac{156543.03392\cos(\phi )}{2^{z}},$$

where *r* is the approximate ground sampling interval in meters per pixel, ϕ is latitude, and *z* is the map level. Using the central latitude of each study region at z=18 gives approximate exported ground sampling intervals of 0.53 m/pixel for Asia, 0.38 m/pixel for Europe, and 0.59 m/pixel for Africa. These values describe the export scale and should not be interpreted as native sensor ground-sampling distances. The year ranges reported above represent the temporal information available during dataset compilation rather than precise acquisition timestamps for every underlying source image. Because the Google basemap interface did not consistently expose the original sensor identity, native spatial resolution, native band metadata, or exact per-scene acquisition timestamp, unavailable metadata were not inferred. The October 2025 date, therefore, denotes basemap access and image retrieval, not the acquisition date of the underlying satellite imagery.

For the North American source-domain subset, NDVI and EVI were generated from the co-registered NAIP red, blue, and near-infrared bands and spatially aligned with the corresponding RGB images. NDVI was calculated as:

(2) $$\mathrm{NDVI}=\frac{NIR-Red}{NIR+Red+\epsilon },$$

and EVI was calculated as:

(3) $$\mathrm{EVI}=2.5\times \frac{NIR-Red}{NIR+6Red-7.5Blue+1+\epsilon },$$

where NIR, Red, and Blue denote the corresponding NAIP spectral bands, and ϵ is a small constant used to avoid division by zero. The resulting index layers were resampled and registered to the same spatial resolution as the RGB imagery before being used by SIAF. GNDVI and SAVI were additionally generated from the North American multispectral data only for the controlled SIAF comparison.

No NDVI, EVI, GNDVI, or SAVI layers were generated for the Asia, Europe, Africa, or FAIS target datasets. In the transfer stage, the source-domain model pretrained with North American RGB, NDVI, and EVI inputs provided the initialization for each target experiment. The target model was then fine-tuned using only the available RGB images and corresponding masks. Therefore, the target-domain results do not depend on unreported or inferred near-infrared information.

To reflect realistic plantation conditions, sample selection follows three principles: regional diversity, temporal variability, and scene complexity. Typical plantation types, particularly fast-growing eucalyptus stands and mixed conifer–broadleaf plantations, are emphasized across diverse climatic and geomorphological settings. Multi-temporal imagery is incorporated to capture cross-scene spectral variation, while challenging scenarios, including cloud and shadow contamination, mountainous terrain, forest–nonforest transition zones, and built-up areas, are deliberately retained to represent practical mapping difficulties.

#### 2.1.1. Plantation Definition and Annotation Protocol

In this study, a plantation was operationally defined as a tree-covered stand established or intensively managed through human planting activities. Plantation areas were identified primarily according to spatial regularity visible in high-resolution imagery, including relatively aligned planting patterns, uniform crown spacing, homogeneous canopy texture, and comparatively clear stand boundaries. These spatial characteristics reflect the regular planting and management patterns commonly associated with plantation stands.

Natural forests were distinguished by their irregular crown distribution, heterogeneous canopy structure, variable tree spacing, and lack of clearly organized planting patterns. Orchards and agroforestry systems were excluded because they generally exhibited agricultural field structures, regularly separated individual tree crowns, intercropping patterns, or close associations with cultivated land and settlements. Shrubs and grasslands were excluded according to their lower canopy height, discontinuous crown structure, and distinct texture characteristics. Mixed conifer–broadleaf stands were included only when regular planting arrangements and identifiable management boundaries indicated a plantation origin. In contrast, mixed forests without reliably distinguishable planting patterns were treated as non-plantation areas. Regions that could not be confidently assigned to either class were excluded from the positive plantation annotations.

Pixel-level plantation masks were manually delineated in Labelme by three annotators with forestry-related backgrounds. The annotators identified plantation regions according to the same operational criteria and delineated boundaries along the visible outer extent of continuous plantation canopies. After the initial annotation stage, all masks were independently reviewed by an additional forestry expert. Disagreements concerning plantation identity, mixed vegetation, or boundary location were resolved by the reviewing expert, whose decision was used as the final annotation.

Internal shadows, forest roads, clearly visible canopy gaps, and other non-canopy areas within plantation stands were excluded from the plantation masks and labeled as background. This annotation and expert-review procedure was consistently applied across all four regional subsets. Because the annotation workflow adopted expert review and consensus adjudication rather than independent repeated annotation of the same complete sample set, no formal inter-annotator agreement statistic was calculated.

**Figure 2:**
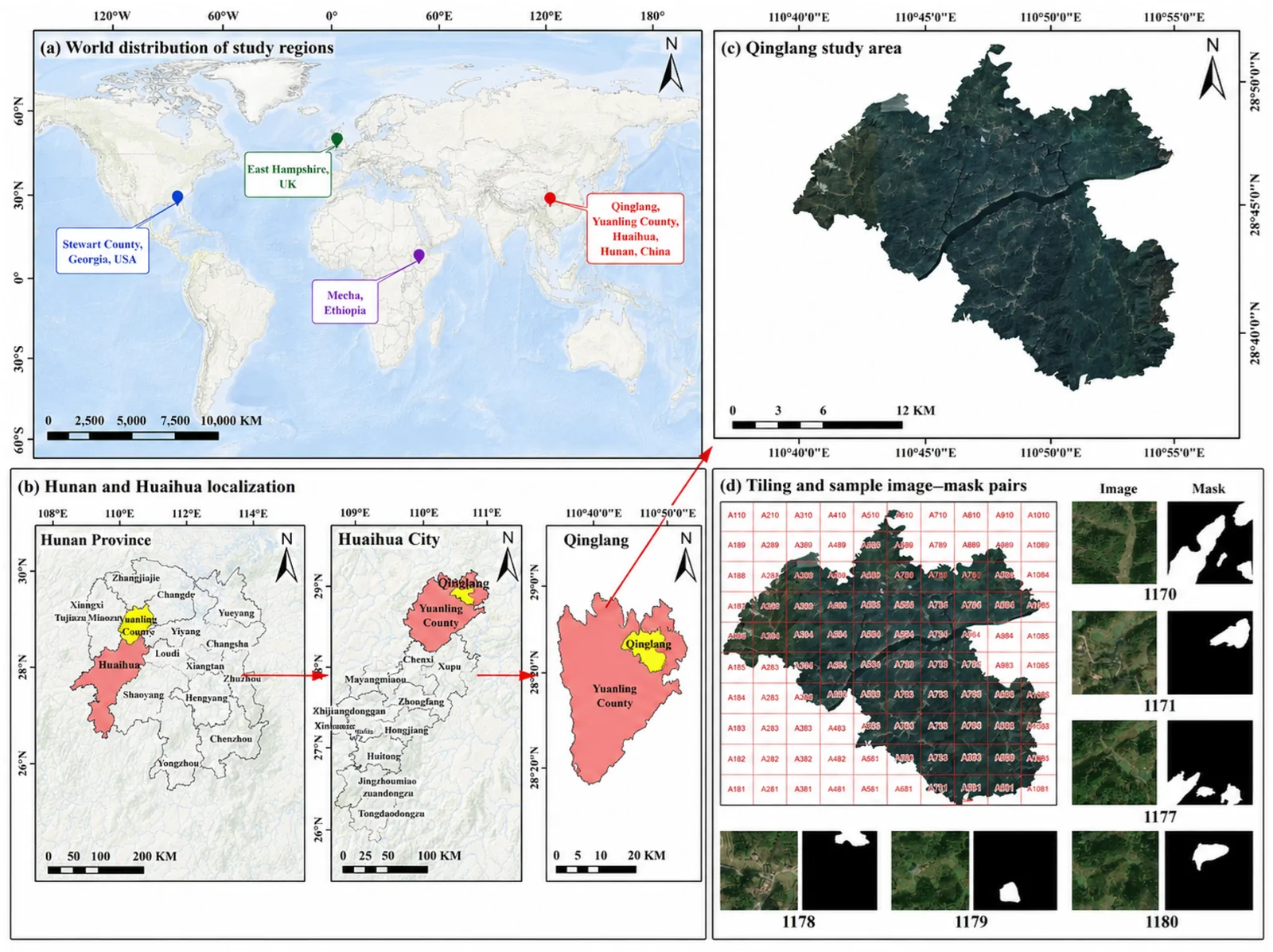
Schematic of dataset acquisition and preprocessing. The left panels show the geographic localization of the representative study area from Hunan Province to Huaihua City and Qinglang region in Yuanling County. The upper-right panel shows the original remote-sensing scene, and the lower-right panel illustrates non-overlapping tiling and representative image–mask pairs.

As illustrated in Figure 2, the North American, Asia, Europe, and Africa subsets were constructed from 24, 23, 22, and 23 original large scenes, respectively, with each scene containing approximately 5000 × 5000 pixels. To prevent both scene-level and geographic multitemporal leakage, partitioning was performed before image tiling using the geographic footprints of the original large scenes. The spatial extents of all original scenes were checked for overlap. Scenes acquired at different times but covering the same or overlapping geographic area were treated as a single geographic group and were required to remain in the same subset. The North American source-domain geographic groups were divided using an approximate ratio of 7:1:2 for training, validation, and testing. In contrast, the Asia, Europe, and Africa target-domain geographic groups were divided using an approximate ratio of 2:1:7, so that only a limited portion of each target region was used for fine-tuning and most target areas remained reserved for independent testing. Non-overlapping 500 × 500-pixel tiles were generated only after this geographic-group-level partitioning. We confirmed that no geographically overlapping multitemporal scenes were assigned to different training, validation, or test subsets. Therefore, neither tiles from the same original scene nor tiles originating from geographically overlapping multitemporal scenes were shared across the data partitions.

All generated tiles and their corresponding masks were subjected to multiple rounds of quality control. Low-quality tiles, empty tiles, samples without useful plantation information, and masks with unresolved annotation ambiguity were removed. The final masks were confirmed by the reviewing forestry expert. After quality control, 1182 image–mask pairs were retained for each region, resulting in 4728 samples across the four continents. The identical sample count across regions was obtained through controlled sampling after quality screening to maintain a balanced regional comparison. These samples provide a high-resolution benchmark for preprocessing, model training, and evaluation.

The North American source-domain subset follows an approximately 70%/10%/20% training–validation–test division, corresponding to 828, 118, and 236 tiles, respectively. The Asia, Europe, and Africa target-domain subsets follow an approximately 20%/10%/70% fine-tuning–validation–test division, corresponding to 236, 118, and 828 tiles per region. The FAIS dataset contains 5108 image–mask pairs [19] and was divided into 1022 fine-tuning samples, 511 validation samples, and 3575 independent test samples. For the self-constructed datasets, geographic-footprint grouping and subset assignment were completed before tiling; all scenes with overlapping geographic coverage, including multitemporal observations of the same area, were kept within a single subset. The exact split lists for all datasets are included in the released repository so that the same samples can be used in reproduction experiments.

#### 2.1.2. Dataset Statistics

To further characterize class composition and the spatial structure of plantation annotations, dataset-level statistics were summarized from the binary ground-truth masks. Plantation and background pixel ratios describe the foreground–background composition of each regional subset. Patch size is summarized from connected plantation components within individual tiles and is therefore interpreted as a tile-level structural descriptor rather than a landscape-scale stand-area estimate. Boundary pixels are defined as the one-pixel inner boundary obtained by subtracting a 3 × 3 eroded plantation mask from the original binary plantation mask, and the boundary-pixel ratio is calculated relative to the total number of plantation pixels. The resulting statistics are summarized in Table 1.

### 2.2. KGF-Net

**Figure 3:**
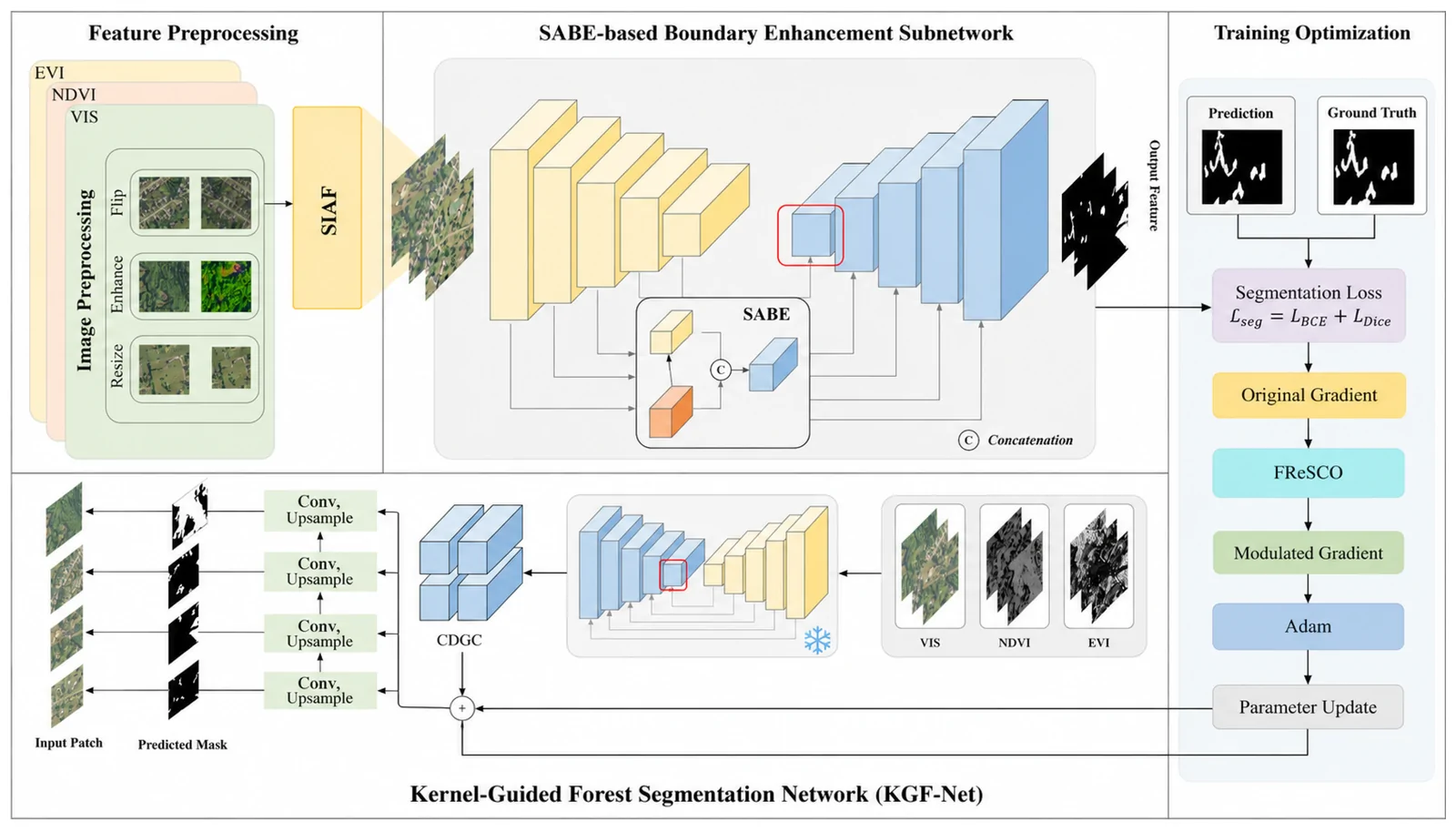
Overall architecture and optimization flow of KGF-Net. During North American source-domain pretraining, RGB imagery and NDVI/EVI layers are aligned by SIAF before the shared backbone, SABE, CDGC, and decoder. During Asia, Europe, Africa, and FAIS adaptation, the transferred model is fine-tuned using target-domain RGB images only. Prediction and ground truth are supervised using $L_{\mathrm{seg}}=L_{\mathrm{BCE}}+L_{\mathrm{Dice}}$. The resulting gradient is modulated by FReSCO before being passed to Adam for parameter updating. No additional auxiliary loss branch is used.

KGF-Net is developed based on the UltraLight-VM-UNet framework [20] and adopts a modular architecture with multi-level enhancements tailored for plantation remote-sensing imagery. As shown in Figure 3, the network consists of four coordinated components: SIAF, SABE, CDGC, and FReSCO. To improve readability, the overall network and the four component designs are presented as separate figures: the main framework is shown at the beginning of this subsection, and the detailed module diagrams are placed in their corresponding methodological subsections.

During North American source-domain pretraining, SIAF integrates high-resolution RGB imagery with NDVI and EVI at a unified spatial scale. The optical branch retains crown arrangement and stand texture, while the vegetation-index branch contributes information related to canopy greenness, density, and condition. SABE is embedded at the encoder–decoder bridge stage to reinforce plantation boundary modeling, and CDGC is incorporated into the decoder to suppress responses from roads, buildings, cropland, bare soil, and other non-plantation backgrounds. FReSCO is applied as an Adam-compatible gradient-modulation strategy during optimization. For RGB-only target-domain adaptation, the shared backbone, SABE, CDGC, decoder, and optical-pathway parameters are initialized from the North American multimodal model, while the target model is fine-tuned without target-domain vegetation-index inputs.

The four components are not interchangeable generic enhancements. Each component targets a distinct source of plantation-segmentation error and introduces a corresponding methodological modification, as summarized in Table 2. SIAF addresses the limited spectral discrimination of RGB-only source training; SABE explicitly models plantation-edge topology rather than relying only on local convolution; CDGC combines channel, spatial, gating, and contextual information rather than applying an isolated attention operation; and FReSCO changes the optimization trajectory through plantation-aware gradient modulation while leaving the inference graph unchanged. Their integration, therefore, forms a complementary spectral–structural–contextual–optimization framework.

During training, KGF-Net is optimized solely using the segmentation objective composed of binary cross-entropy loss and Dice loss:

(4) $$L_{\mathrm{seg}}=L_{\mathrm{BCE}}+L_{\mathrm{Dice}}.$$

No additional KL-divergence, reconstruction, quantization, alignment, or boundary-consistency loss is used in the reported experiments. FReSCO operates on the gradient generated from $L_{\mathrm{seg}}$ and modulates this gradient before the Adam parameter update; therefore, it does not introduce an additional optimization objective. Owing to its compact architecture and coordinated module design, KGF-Net is well suited for fine-grained plantation segmentation across diverse geographic regions and imaging conditions.

### 2.3. SIAF

**Figure 4:**
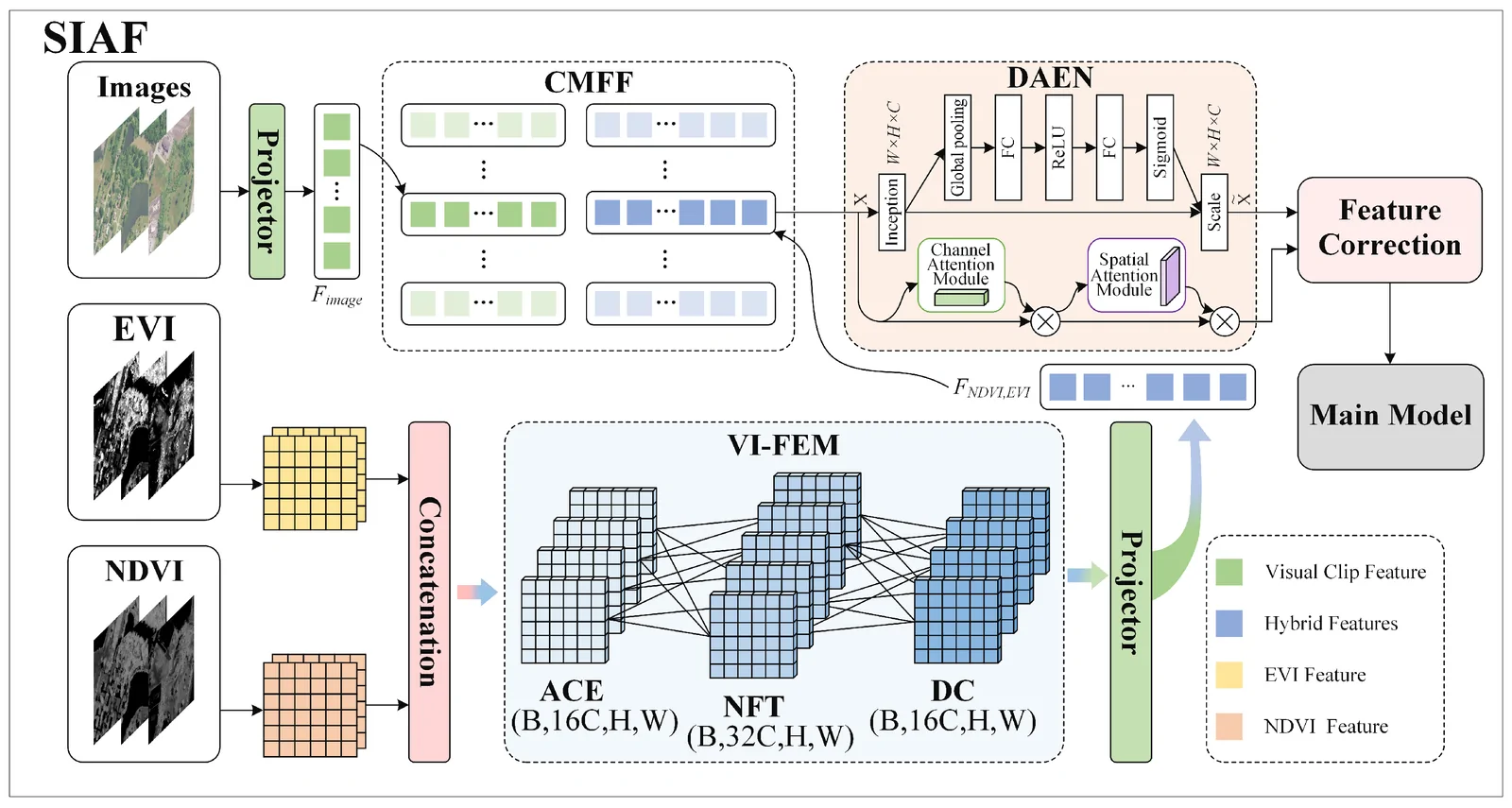
Detailed structure of SIAF for source-domain multimodal feature fusion. RGB and vegetation-index features are integrated to learn transferable representations for RGB-only target-domain adaptation.

The design of SIAF is motivated by the complementary characteristics of high-resolution optical imagery and vegetation indices. In this study, SIAF is used during North American source-domain pretraining, where optical features provide fine-grained spatial structure and NDVI/EVI provide vegetation-sensitive spectral information. As shown in Figure 4, SIAF aligns these source-domain representations at the same spatial scale and constructs a multimodal feature space whose shared parameters are subsequently transferred to RGB-only target-domain fine-tuning. The module follows a sequential pipeline consisting of vegetation-index feature extraction (VI-FEM), cross-modal feature fusion (CMFF), dual-attention enhancement (DAEN), and feature correction.

Given an input optical image, visual features are first extracted and projected into a compact representation $F_{\mathrm{image}}$. In parallel, NDVI and EVI maps are concatenated and fed into VI-FEM, which adopts a sandglass-shaped architecture composed of adaptive channel expansion (ACE), nonlinear feature transformation (NFT), and dimensionality compression (DC). This process produces a compact but spectrally sensitive vegetation-index feature map $F_{\mathrm{NDVI},\mathrm{EVI}}$.

Subsequently, CMFF aligns and fuses $F_{\mathrm{image}}$ and $F_{\mathrm{NDVI},\mathrm{EVI}}$ at the same spatial scale through cross-modal projection and normalization, yielding hybrid multimodal features. The fused representation is then refined by DAEN, which integrates channel attention and spatial attention to enhance discriminative responses while preserving boundary continuity. Finally, a feature correction operation rescales the output to ensure numerical stability before feeding it into the main network.

The complete formulation of SIAF is summarized as follows:

(5) $$F_{\mathrm{NDVI},\mathrm{EVI}}=\mathrm{DC}\left(\phi \left(\mathrm{ACE}\left([I_{\mathrm{NDVI}},I_{\mathrm{EVI}}]\right)\right)\right),$$

(6) $$F_{\mathrm{fusion}}=N\left([F_{\mathrm{image}},F_{\mathrm{NDVI},\mathrm{EVI}}]\right),$$

(7) $$w_{c}=\sigma \left({\mathrm{FC}}_{2}\left(\delta \left({\mathrm{FC}}_{1}\left(\mathrm{GAP}(F_{\mathrm{fusion}})\right)\right)\right)\right),$$

(8) $$w_{s}=\sigma \left(\mathrm{Conv}\left([\mathrm{AvgPool}\left(F_{\mathrm{fusion}}\right),\mathrm{MaxPool}\left(F_{\mathrm{fusion}}\right)]\right)\right),$$

(9) $$F_{\mathrm{out}}=\mathrm{Scale}\left(F_{\mathrm{fusion}}⊗w_{c}⊗w_{s}\right),$$

where $I_{\mathrm{NDVI}}$ and $I_{\mathrm{EVI}}$ denote the NDVI and EVI maps, respectively; ϕ(·) denotes the nonlinear feature transformation; N(·) denotes the normalization operation; σ(·) denotes the sigmoid activation function; δ(·) denotes the ReLU activation function; GAP(·) denotes global average pooling; $w_{c}$ and $w_{s}$ denote the channel and spatial attention weights, respectively; and ⊗ denotes element-wise multiplication.

Through the above pipeline, SIAF establishes an explicit correspondence between optical features and vegetation-index representations in the source domain. The resulting multimodal pretraining weights are transferred to the shared segmentation network, after which each target-domain model is adapted using RGB images only.

### 2.4. SABE

Plantation boundaries in high-resolution remote-sensing imagery are often fragmented and irregular due to canopy overlap, shelterbelts, terrain variation, and interference from surrounding vegetation. Conventional convolution-based feature extraction lacks explicit structural constraints and therefore tends to produce blurred or locally missing boundaries in such scenarios. To address this issue, we propose SABE, a structure-aware boundary enhancement module that explicitly models boundary relationships while maintaining adaptability to complex semantic variations.

**Figure 5:**
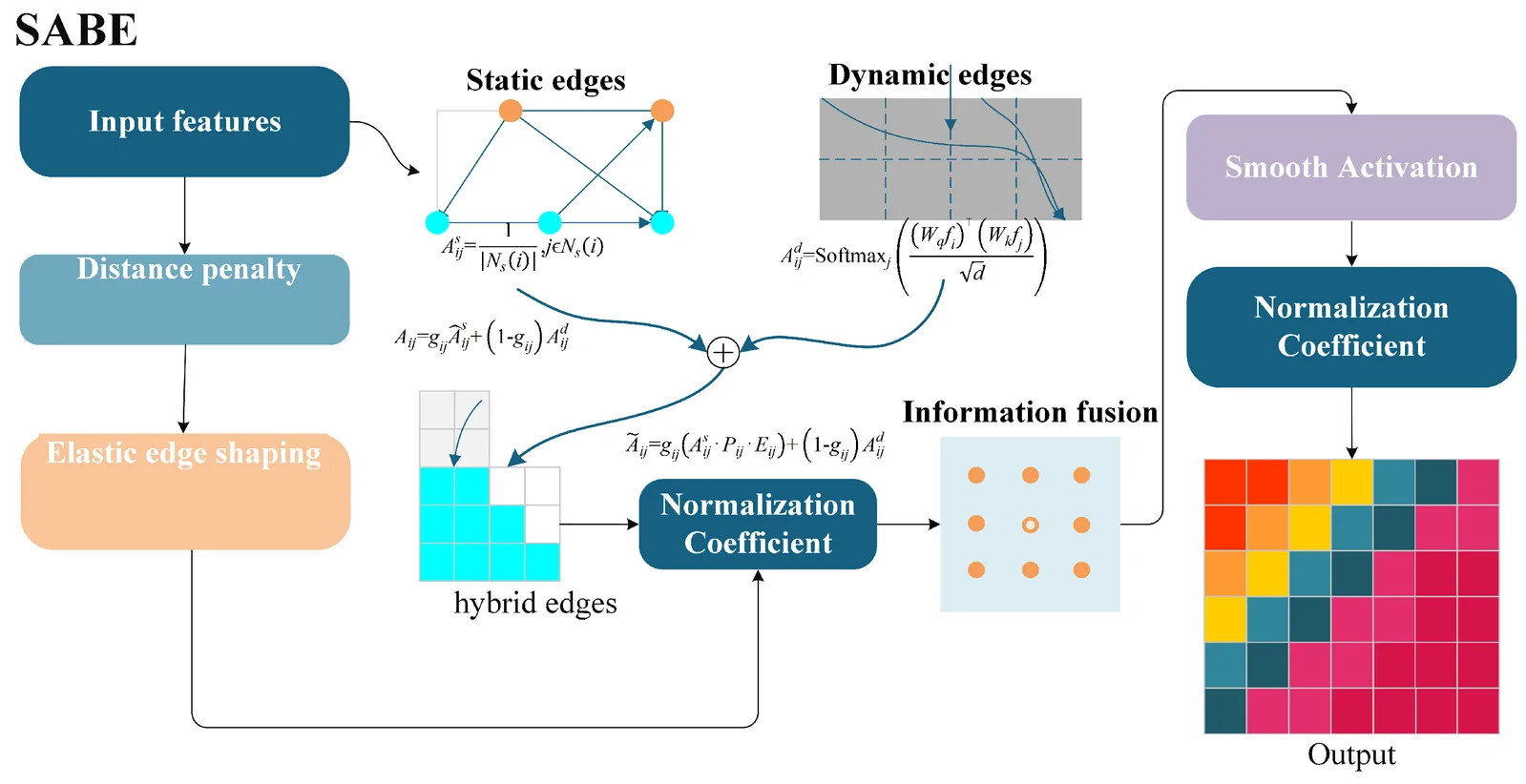
Detailed structure of the structure-aware boundary enhancement module (SABE). SABE integrates static geometric adjacency and dynamic semantic adjacency for boundary-aware feature enhancement.

As shown in Figure 5, SABE constructs boundary-aware representations by jointly exploiting static geometric adjacency and dynamic semantic adjacency. Static adjacency is defined on the regular image grid and provides stable local structural priors, which help preserve boundary continuity and topological consistency. Dynamic adjacency is learned from feature similarity and captures adaptive semantic relationships among neighboring regions, enabling the model to respond to local appearance variations along plantation edges. By combining these two complementary forms of adjacency, SABE balances structural regularity and semantic flexibility in boundary modeling.

To make the structural branch more consistent with boundary geometry, we further introduce a distance penalty term and an elastic edge-weight shaping term. The distance penalty is defined as:

(10) $$P_{ij}=\exp\left(-\frac{{\left|p_{i}-p_{j}\right|}_{2}^{2}}{\sigma^{2}}\right),$$

where $p_{i}$ and $p_{j}$ denote the spatial coordinates of nodes *i* and *j*, respectively, and *σ* controls the spatial decay range. This term suppresses structurally implausible long-range interactions.

The elastic edge-weight shaping term is formulated as:

(11) $$E_{ij}=1+\lambda \cdot \tanh\left(\left|b_{i}-b_{j}\right|\right),$$

where $b_{i}$ and $b_{j}$ denote local boundary-response scores, and *λ* is a scaling factor. This term enhances boundary-relevant interactions while avoiding excessive amplification in homogeneous regions.

To further regulate the interaction between static and dynamic relations, SABE introduces an adaptive gating mechanism that modulates their relative contributions according to local feature characteristics. This mechanism allows the model to rely more on geometric constraints in structurally homogeneous boundary regions, while emphasizing semantic relations in areas affected by texture perturbations or background interference. Accordingly, the refined boundary-aware affinity can be written as:

(12) $${\tilde{A}}_{ij}=g_{ij}\left(A_{ij}^{s}\cdot P_{ij}\cdot E_{ij}\right)+\left(1-g_{ij}\right)A_{ij}^{d},$$

where $A_{ij}^{s}$ and $A_{ij}^{d}$ denote the static geometric adjacency and dynamic semantic adjacency, respectively, and $g_{ij}$ denotes the adaptive gating coefficient.

In addition, SABE adopts a multi-branch aggregation strategy to integrate self-information and neighborhood information from different relational perspectives, thereby enhancing robustness to fragmented edges and small-scale discontinuities. Through the above design, SABE effectively strengthens boundary awareness and preserves the geometric integrity of plantation outlines under complex conditions.

### 2.5. CDGC

**Figure 6:**
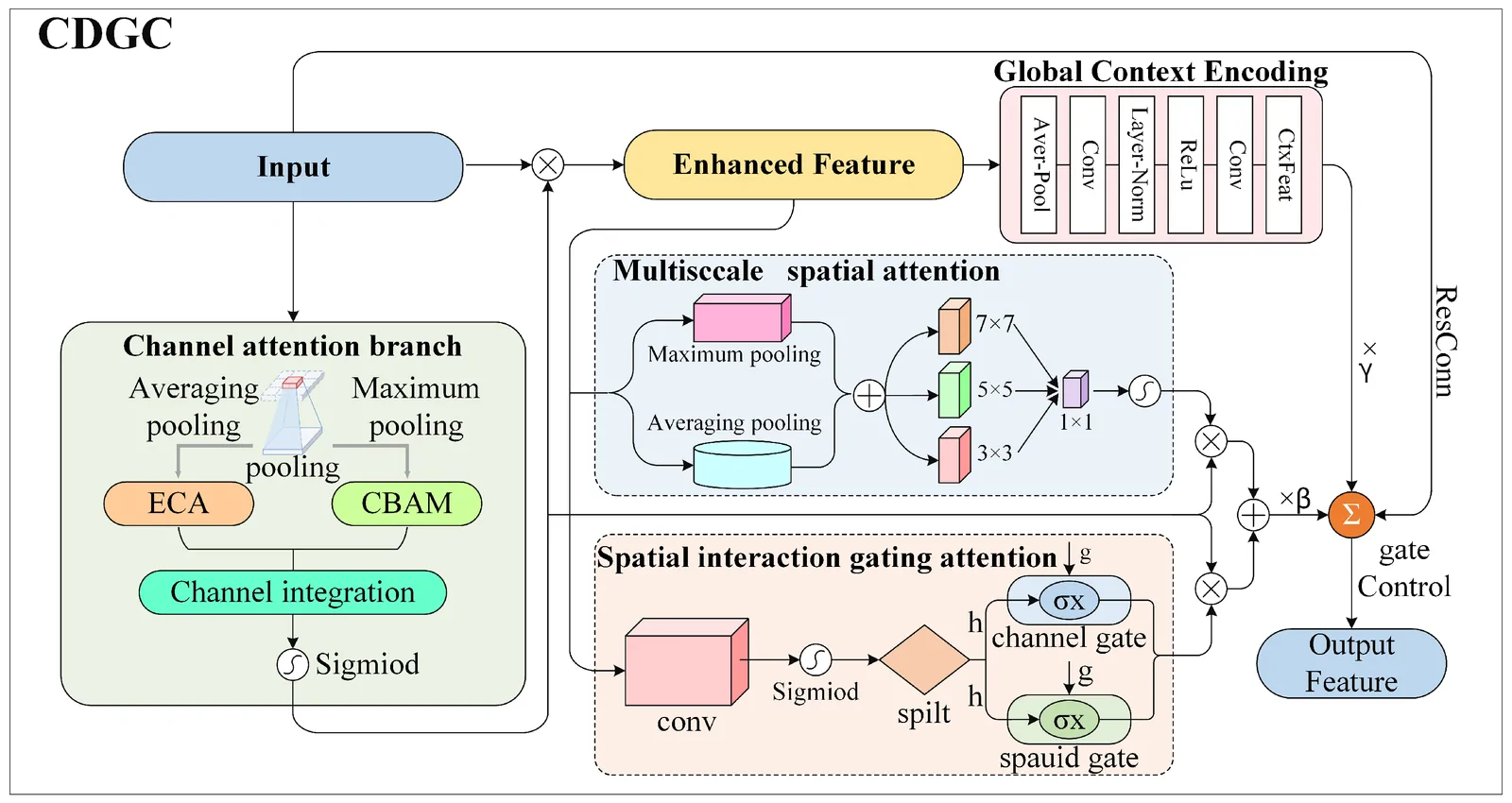
Detailed structure of the cross-domain discriminative geometry-constrained coupling module (CDGC). CDGC integrates channel, spatial, and contextual features for cross-domain feature enhancement.

Inspired by the observation that plantation canopies exhibit relatively stable geometric layouts while background objects such as buildings, roads, bare soil, and grassland introduce irregular and heterogeneous responses in high-resolution imagery, we design CDGC to explicitly regulate feature responses across channel, spatial, and contextual dimensions. As illustrated in Figure 6, CDGC follows a cooperative attention and gating paradigm to enhance canopy-related activations while suppressing background interference.

The input feature is first processed by a channel attention branch, where complementary mechanisms are jointly employed to recalibrate channel-wise responses. By integrating lightweight channel attention and channel–spatial attention, this branch enhances discriminative spectral responses associated with plantation canopies while attenuating channels dominated by background materials with similar spectral characteristics.

Subsequently, the channel-refined feature is fed into a multi-scale spatial attention module, which captures spatial dependencies at different receptive-field scales through parallel convolutions. This design allows the model to adaptively respond to plantations of varying sizes and shapes, while preserving sensitivity to slender shelterbelts and fragmented canopy structures commonly observed in plantation scenes.

To further regulate the interaction between channel and spatial cues, CDGC introduces a spatial interaction gating mechanism, which generates adaptive gating signals and decomposes them into channel-wise and spatial-wise components. These gates selectively control information flow from different attention branches, preventing excessive amplification of background regions and stabilizing feature responses near plantation boundaries.

In addition, a global context encoding module aggregates scene-level information via global pooling and lightweight transformations. This global representation complements local attention by providing contextual cues for resolving ambiguities in highly mixed land-cover regions. Finally, the original input feature, attention-enhanced feature, and global context feature are fused through residual connections to produce the output feature.

Through coordinated channel discrimination, multi-scale spatial modeling, adaptive gating, and global context integration, CDGC provides more stable discriminative capability for plantation segmentation and effectively prevents mis-segmentation under complex background conditions.

### 2.6. FReSCO

The design of FReSCO is motivated by the observation that plantation segmentation in remote-sensing imagery is often affected by boundary ambiguity, texture heterogeneity, spectral imbalance, and scale variation across regions and acquisition conditions. These factors may lead to unstable or insufficient gradient updates when a conventional optimization strategy is directly applied. Rather than serving as an independent optimizer or modifying the network architecture, FReSCO is designed as a forest-aware gradient-modulation strategy implemented on top of Adam.

**Figure 7:**
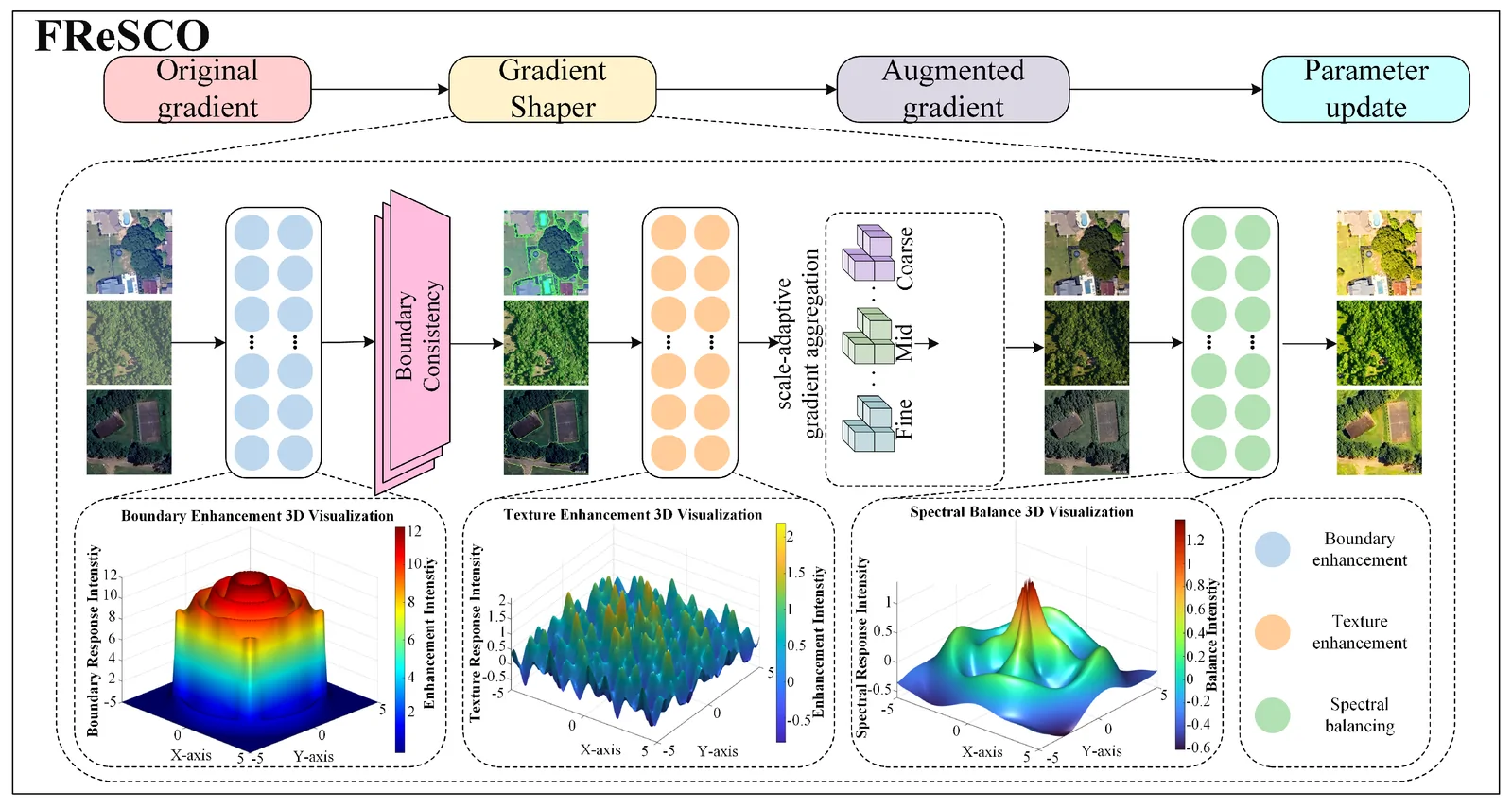
Detailed structure of the forest-aware responsive spectral control strategy (Adam + FReSCO). FReSCO modulates optimization gradients during training to improve forest-aware feature learning.

In all experiments reported in this study, FReSCO is used together with Adam and should therefore be interpreted as Adam + FReSCO. As illustrated in Figure 7, FReSCO receives the gradient generated by the segmentation objective, estimates four plantation-related modulation factors from the current mini-batch, and rescales the gradient before the Adam parameter update. No additional loss term or inference-time branch is introduced.

Let $\theta_{t}$ denote the trainable parameters at iteration *t*. Given the segmentation objective $L_{\mathrm{seg}}$, the original gradient is

(13) $$g_{t}=\nabla_{\theta }L_{\mathrm{seg}}\left(\theta_{t}\right).$$

Four normalized mini-batch-level terms are then calculated. The boundary-aware term measures prediction errors around plantation boundaries extracted from the ground-truth mask; the texture-aware term measures normalized local intensity variation; the spectral-balance term measures channel-level intensity imbalance; and the scale-aware term measures variation in plantation occupancy among samples. Their calculations are summarized as

(14) $$\begin{array}{cc} M_{t}^{b} & =2\frac{\sum B_{t}⊙\left|P_{t}-Y_{t}\right|}{\sum B_{t}+\epsilon }-1,\\ M_{t}^{\mathrm{tex}} & =2\mathrm{Mean}\left(\frac{V_{t}-\min\left(V_{t}\right)}{\max\left(V_{t}\right)-\min\left(V_{t}\right)+\epsilon }\right)-1,\\ M_{t}^{\mathrm{spec}} & =2\mathrm{clip}\left(\frac{{\mathrm{Std}}_{c}\left(\mu_{c}\right)}{{\mathrm{Mean}}_{c}\left(\right|\mu_{c}\left|\right)+\epsilon },0,1\right)-1,\\ M_{t}^{\mathrm{scale}} & =2\mathrm{clip}\left(\frac{{\mathrm{Std}}_{n}\left(a_{n}\right)}{{\mathrm{Mean}}_{n}\left(a_{n}\right)+\epsilon },0,1\right)-1. \end{array}$$

Here, $P_{t}$ and $Y_{t}$ denote the predicted probability map and ground-truth mask, respectively; $B_{t}$ is the binary boundary map obtained using a morphological gradient of $Y_{t}$; $V_{t}$ is the 3 × 3 local variance map of the grayscale input image; $\mu_{c}$ is the mini-batch mean intensity of channel *c*; and $a_{n}$ denotes the plantation-pixel proportion of sample *n*. During North American source-domain pretraining, the spectral term is calculated using RGB, NDVI, and EVI inputs, whereas during target-domain adaptation it is calculated from RGB channels only.

The four terms are combined into a single gradient-modulation coefficient:

(15) $$M_{t}=\mathrm{clip}\left(1+\alpha_{b}M_{t}^{b}+\alpha_{\mathrm{tex}}M_{t}^{\mathrm{tex}}+\alpha_{\mathrm{spec}}M_{t}^{\mathrm{spec}}+\alpha_{\mathrm{scale}}M_{t}^{\mathrm{scale}},M_{\min},M_{\max}\right).$$

The coefficients are set to $\alpha_{b}=0.30$, $\alpha_{\mathrm{tex}}=0.20$, $\alpha_{\mathrm{spec}}=0.20$, and $\alpha_{\mathrm{scale}}=0.15$. The modulation coefficient is clipped to $\left[M_{\min},M_{\max}\right]=\left[0.50,1.50\right]$ to prevent excessive gradient suppression or amplification.

The resulting coefficient is applied to the original gradient:

(16) $${\tilde{g}}_{t}=M_{t}g_{t},$$

and Adam subsequently updates the model parameters using the modulated gradient:

(17) $$\theta_{t+1}=\mathrm{Adam}\left(\theta_{t},{\tilde{g}}_{t};\eta ,\beta_{1},\beta_{2}\right),$$

where *η* denotes the learning rate and $\beta_{1}$ and $\beta_{2}$ are the Adam momentum coefficients.

Accordingly, FReSCO follows four operations in each training iteration: the segmentation loss is first back-propagated to obtain the original gradient; boundary-, texture-, spectral-, and scale-aware statistics are then estimated from the current mini-batch; these statistics are combined into a bounded modulation coefficient; and the resulting gradient is passed to Adam for parameter updating. The boundary term emphasizes difficult plantation-edge regions, the texture term responds to local image heterogeneity, the spectral term compensates for channel-level imbalance, and the scale term adjusts the update according to differences in plantation occupancy.

During source-to-target transfer, the shared backbone, SABE, CDGC, decoder, and RGB optical-pathway parameters are initialized from the North American source-domain checkpoint. Parameters associated exclusively with the vegetation-index input pathway are not used in the RGB-only target model. FReSCO itself introduces no independently learned network parameters; the same gradient-modulation procedure is reapplied during RGB-only target-domain fine-tuning using the statistics available from the target RGB mini-batches.

Therefore, FReSCO modifies the optimization trajectory without changing the segmentation objective or inference architecture. It provides plantation-aware gradient regulation while retaining the standard Adam update mechanism and introduces no additional computational branch during inference.

## 3. Results

### 3.1. Experimental Environment and Training Details

To comprehensively evaluate the segmentation performance and computational efficiency of KGF-Net for plantation mapping, four groups of experiments were conducted. First, module-effectiveness experiments were performed on the North American source-domain benchmark to evaluate SIAF, SABE, CDGC, and FReSCO. Second, systematic ablation experiments were conducted on the same source-domain subset. Third, comparative experiments were carried out against representative semantic segmentation models under a unified evaluation protocol. Fourth, cross-region transfer experiments were conducted on the Asia, Europe, Africa, and public FAIS target datasets. The source model was pretrained on North America using RGB, NDVI, and EVI with an approximately 70%/10%/20% split. Each target model was then fine-tuned using only approximately 20% of the target RGB data, validated on 10%, and evaluated on the remaining 70%.

For the controlled module-effectiveness experiments, a one-factor-at-a-time protocol was adopted. The SIAF fusion comparison was conducted under the complete Group I configuration in Table 9, with SABE, CDGC, and FReSCO kept fixed while the fusion strategy and vegetation-index input were varied.

The SABE replacement experiment was conducted under the fixed Group G configuration in Table 9, namely SIAF + SABE + FReSCO without CDGC. In this comparison, only SABE was replaced by the corresponding graph-based alternative, while SIAF, FReSCO, the backbone architecture, dataset split, loss function, input size, and other training settings were kept unchanged.

The CDGC replacement experiment was conducted under the fixed Group H configuration, namely SIAF + SABE + CDGC without FReSCO. Only CDGC was replaced by the corresponding attention or feature-reweighting module, while all remaining components and experimental settings were fixed.

The optimization-strategy comparison was conducted under the fixed Group F configuration, namely SIAF + CDGC + FReSCO without SABE. In this experiment, the network architecture was kept unchanged, and only the optimization strategy was varied.

This design ensured that each controlled comparison changed only the component or optimization strategy under evaluation, allowing the observed performance differences to be attributed primarily to the corresponding factor.

All models were implemented using PyTorch and trained on a workstation equipped with an AMD Ryzen 7 5800H CPU (Advanced Micro Devices, Inc., Santa Clara, CA, USA) and a single NVIDIA GeForce RTX 3090 GPU (NVIDIA Corporation, Santa Clara, CA, USA) with 24 GB of video memory. The detailed hardware and software environment is summarized in Table 3.

North American source-domain pretraining used three RGB channels together with two spatially aligned vegetation-index channels corresponding to NDVI and EVI. Asia, Europe, Africa, and FAIS target-domain fine-tuning used three RGB channels only. The training batch size was set to 8, whereas the validation and test batch sizes were set to 1. The North American source model was trained for 300 epochs, and the transferred target models were fine-tuned under the same epoch budget and checkpoint-selection rule.

The training objective was defined as an equally weighted combination of binary cross-entropy loss and Dice loss:

(18) $$L_{\mathrm{seg}}=L_{\mathrm{BCE}}+L_{\mathrm{Dice}},$$

where $L_{\mathrm{BCE}}$ provides pixel-wise binary supervision and $L_{\mathrm{Dice}}$ alleviates class imbalance by maximizing the overlap between the predicted plantation mask and the reference mask. Both loss weights were set to 1. No KL-divergence loss, reconstruction loss, quantization loss, alignment loss, or auxiliary boundary-consistency loss was used in the reported training procedure.

The complete KGF-Net was trained using Adam-compatible FReSCO gradient modulation. The underlying Adam parameters were set to an initial learning rate of 1 × 10^−3^, momentum coefficients of $\beta_{1}=0.9$ and $\beta_{2}=0.999$, numerical-stability constant $\epsilon =1\times 10^{-8}$, and weight decay of 1 × 10^−2^. The learning rate was controlled using a cosine-annealing scheduler with $T_{\max}=50$ and a minimum learning rate of 1 × 10^−5^. The scheduler was updated once after each training epoch.

During training, data augmentation was applied to improve robustness to orientation, scale, illumination, and geometric variation. The augmentation operations included horizontal flipping, vertical flipping, random $90^{\circ }$ rotation, shift–scale–rotation transformation, random brightness and contrast adjustment, elastic deformation, grid distortion, and optical distortion. Horizontal flipping, vertical flipping, random $90^{\circ }$ rotation, shift–scale–rotation, and brightness–contrast adjustment were each applied with a probability of 0.5. The shift limit, scale limit, and rotation limit were set to 0.0625, 0.1, and $45^{\circ }$, respectively. One of the elastic, grid, or optical distortion operations was additionally selected with an overall probability of 0.3.

The random seed was fixed at 42 for Python, NumPy, and PyTorch. Deterministic cuDNN execution was enabled and cuDNN benchmarking was disabled to reduce nondeterministic variation. Mixed-precision training was not used, and all reported experiments were performed using full-precision floating-point computation. Because the experiments were conducted with a single fixed random seed, all between-method and between-configuration differences reported below are interpreted as descriptive numerical rankings under this protocol. They are not treated as estimates of run-to-run variability or as evidence of statistically established superiority.

Linear-layer weights were initialized using a truncated normal distribution with a standard deviation of 0.02, while convolutional layers were initialized using variance-scaled normal initialization according to their kernel size and output-channel number. Bias terms were initialized to zero. No externally trained segmentation checkpoint was used for KGF-Net or any comparison model in the North American source-domain experiments; all source-domain models were trained from their implementation-level initialization under the unified protocol described above. Each Asia, Europe, Africa, and FAIS target-domain model was initialized only from the corresponding North American source-domain checkpoint produced in this study. Therefore, no method received additional target-domain pretrained weights.

Model performance was evaluated on the validation subset after every epoch, while the complete evaluation metrics were recorded every 10 epochs. The checkpoint with the lowest validation loss was retained as the final model and subsequently evaluated on the independent test subset. Early stopping was not used. During evaluation, prediction probabilities were converted into binary masks using a fixed threshold of 0.5.

For the cross-region transfer experiments, the North American subset was treated as the source domain. KGF-Net was first pretrained using North American RGB, NDVI, and EVI inputs with an approximately 70%/10%/20% training–validation–test split. For each target dataset, the shared model parameters and RGB optical pathway were initialized from this source checkpoint, while the target-domain vegetation-index branch was not used. Approximately 20% of the target RGB data were used for fine-tuning, 10% were used for validation and checkpoint selection, and the remaining 70% were reserved for final evaluation. These experiments therefore assess multimodal source pretraining followed by limited RGB-based target adaptation, rather than zero-shot generalization or independent multimodal target training.

The principal training settings are summarized in Table 4.

For all ablation and component-replacement experiments, the same scene-level data split, input resolution, augmentation policy, loss function, epoch budget, and checkpoint-selection criterion were used. For comparisons with baseline segmentation models, all methods were trained and evaluated using the same scene-level training, validation, and test subsets and the same binary evaluation threshold, thereby preventing differences in data partitioning from affecting the comparison results. On the North American benchmark, single-modality baselines used RGB inputs, whereas multimodal baselines used RGB as the primary modality and NDVI/EVI as aligned auxiliary modalities, matching the information available to KGF-Net. No separate exhaustive hyperparameter search was conducted for any individual method. Shared optimization and data-processing settings were fixed across models, while architecture-specific settings not covered by the shared protocol followed the corresponding public implementation. This prevented any baseline or KGF-Net from receiving a larger tuning budget.

### 3.2. Evaluation Metrics

To comprehensively evaluate plantation segmentation performance, five general region-level metrics are adopted, including mean Intersection over Union (mIoU), Accuracy, Specificity, Sensitivity, and F1-score. All five metrics are calculated from the confusion matrix, where TP, TN, FP, and FN denote true positives, true negatives, false positives, and false negatives, respectively. Plantation is treated as the positive class. Confusion-matrix counts are accumulated over all pixels in the complete test subset before metric calculation, rather than averaged independently across image tiles. The reported mIoU is the arithmetic mean of plantation-class IoU and background-class IoU, whereas F1-score, Sensitivity, and Specificity are reported with respect to the plantation-positive binary formulation. Consequently, converting the reported F1-score to IoU yields the plantation-class IoU and not the reported two-class mIoU. In addition, because SABE is specifically designed for boundary modeling, Boundary F1-score (BF1) is reported as an additional dedicated boundary metric in the SABE effectiveness experiment. All metric values are rounded to two decimal places after calculation.

Mean Intersection over Union (mIoU) measures the overlap between the predicted plantation regions and the ground-truth labels. It is used as the main indicator of segmentation accuracy. In scenes with complex plantation boundaries, a higher mIoU indicates better regional consistency and more accurate boundary localization. It is defined as:

(19) $$\mathrm{mIoU}=\frac{1}{C}\sum_{c=1}^{C}\frac{{\mathrm{TP}}_{c}}{{\mathrm{TP}}_{c}+{\mathrm{FP}}_{c}+{\mathrm{FN}}_{c}},$$

where *C* denotes the number of classes, and ${\mathrm{TP}}_{c}$, ${\mathrm{FP}}_{c}$, and ${\mathrm{FN}}_{c}$ denote the true positives, false positives, and false negatives of class *c*, respectively.

Accuracy measures the proportion of correctly classified pixels among all pixels. It is calculated as:

(20) $$\mathrm{Accuracy}=\frac{\mathrm{TP}+\mathrm{TN}}{\mathrm{TP}+\mathrm{TN}+\mathrm{FP}+\mathrm{FN}}.$$

Specificity measures the proportion of correctly predicted non-plantation pixels among all actual non-plantation pixels. This metric is especially important in scenes with complex backgrounds, where bare soil, buildings, roads, and grassland may be incorrectly segmented as plantations. It is defined as:

(21) $$\mathrm{Specificity}=\frac{\mathrm{TN}}{\mathrm{TN}+\mathrm{FP}}.$$

Sensitivity measures the ability of the model to identify plantation regions. It is particularly important for small, fragmented, or discontinuous plantation patches. It is calculated as:

(22) $$\mathrm{Sensitivity}=\frac{\mathrm{TP}}{\mathrm{TP}+\mathrm{FN}}.$$

F1-score is the harmonic mean of Precision and Sensitivity. It provides a balanced evaluation of segmentation correctness and completeness, especially when plantations and surrounding natural vegetation are difficult to distinguish. It is defined as:

(23) $$F1=\frac{2\times \mathrm{Precision}\times \mathrm{Sensitivity}}{\mathrm{Precision}+\mathrm{Sensitivity}},\;\mathrm{Precision}=\frac{\mathrm{TP}}{\mathrm{TP}+\mathrm{FP}}.$$

Boundary F1-score (BF1) is additionally used in the SABE effectiveness experiment to quantify plantation-boundary delineation. Let $B_{P}$ and $B_{G}$ denote the boundary-pixel sets extracted from the predicted mask and ground-truth mask, respectively, and let $D_{\tau }\left(\cdot \right)$ denote morphological dilation with a tolerance of *τ* pixels. Boundary precision and boundary recall are defined by matching a predicted or reference boundary pixel when a corresponding boundary pixel lies within the tolerance band:

(24) $$P_{b}=\frac{\left|B_{P}\cap D_{\tau }\left(B_{G}\right)\right|}{\left|B_{P}\right|},\;R_{b}=\frac{\left|B_{G}\cap D_{\tau }\left(B_{P}\right)\right|}{\left|B_{G}\right|},\;\mathrm{BF}1=\frac{2P_{b}R_{b}}{P_{b}+R_{b}}.$$

In this study, τ=2 pixels is used on the prediction masks. Boundary matches are accumulated over the complete North American test subset before calculating BF1. A higher BF1 indicates more accurate and spatially consistent plantation-boundary delineation.

During evaluation, the predicted probability maps and ground-truth labels are binarized using a threshold of 0.5. The five general metrics quantify regional overlap, background suppression, and plantation recall, while BF1 provides an additional boundary-specific assessment for the controlled SABE comparison.

### 3.3. Module Effectiveness Experiments

This section evaluates the effectiveness of the four proposed components, namely SIAF, SABE, CDGC, and FReSCO. For each component, representative alternative methods are used for comparison under controlled settings. In all effectiveness experiments, only the target component is changed, while the remaining network architecture, training strategy, and evaluation protocol are kept unchanged. This one-factor-at-a-time protocol ensures that the performance variation mainly comes from the component being evaluated.

#### 3.3.1. Effectiveness of SIAF

SIAF is evaluated on the North American source-domain benchmark, where multispectral NAIP data permit the generation of vegetation-index inputs. To examine the contribution of different vegetation indices and fusion mechanisms, VIS imagery is used as the baseline input and GNDVI, SAVI, EVI, NDVI, and NDVI+EVI are compared. Four fusion strategies are evaluated under the same source-domain settings: channel concatenation, FiLM [21], a dual-stream fusion baseline adapted from the boundary-enhanced dual-stream network [22], and SIAF. These experiments characterize the source-domain representation learned before RGB-only target adaptation.

As shown in Table 5, the vegetation-index settings yield higher numerical segmentation metrics than the VIS-only baseline under the fixed-seed protocol. Among the single-index settings, EVI and NDVI generally record larger numerical gains than GNDVI and SAVI. The combined NDVI+EVI setting records the highest overall numerical results across the evaluated fusion strategies, which is consistent with complementary information from the two indices under this experimental configuration.

The design of the fusion mechanism is also associated with different numerical outcomes. Under all vegetation-index configurations, SIAF records the highest numerical mIoU and generally higher F1-score, Accuracy, and Specificity than channel concatenation, FiLM, and dual-stream fusion, although the highest numerical Sensitivity is obtained by FiLM under the VIS+NDVI+EVI setting. With VIS+NDVI+EVI input, SIAF records 69.36% mIoU, 91.43% F1-score, 86.72% Accuracy, 96.66% Specificity, and 84.76% Sensitivity. Relative to the VIS-only baseline, the observed mIoU difference is 5.48 percentage points. Relative to the dual-stream fusion strategy under the same VIS+NDVI+EVI input, the observed mIoU difference is 1.22 percentage points. These differences are reported as fixed-seed numerical comparisons rather than statistically established improvements.

Under the specified protocol, the joint VIS+NDVI+EVI configuration is associated with higher source-domain numerical metrics than VIS alone. The resulting source-domain representation is subsequently transferred to RGB-only target-domain models. To further isolate whether vegetation-index-assisted source pretraining contributes to target-domain adaptation, Section 3.6 additionally compares random initialization, RGB-only source pretraining, and RGB+NDVI+EVI source pretraining under the same RGB-only target adaptation protocol.

#### 3.3.2. Effectiveness of SABE

To compare the proposed SABE design with generic graph convolution operators, we replace SABE with several representative graph-based modules at the same bridge position, including GCN [23], APPNP [24], ChebConv [25], GATConv [26], Gated GraphConv [27], GINConv [28], SAGEConv [29], and TAGConv [30]. During this comparison, only the graph-related module is changed, while the remaining model architecture and training settings are kept the same. Because SABE is specifically designed to enhance plantation boundaries, BF1 is additionally reported in this controlled comparison to provide a direct quantitative assessment of boundary-delineation quality. The results are summarized in Table 6.

As shown in Table 6, SABE records the highest numerical mIoU, F1-score, Accuracy, and BF1 under the fixed-seed protocol, reaching 67.92%, 90.77%, 85.78%, and 83.64%, respectively. SAGEConv records the highest numerical Specificity of 96.51%, whereas ChebConv records the highest numerical Sensitivity of 84.01%. SABE records Specificity and Sensitivity values of 96.46% and 83.67%, respectively. For the dedicated boundary metric, the observed BF1 difference between SABE and SAGEConv, the second-highest numerical result, is 0.92 percentage points. This fixed-seed boundary ranking is consistent with the intended structural boundary-enhancement role of SABE, but it is not interpreted as evidence of statistical superiority.

In addition, the placement of SABE also affects both accuracy and computational cost. When SABE is moved from the bridge stage to shallower high-resolution features, the model may obtain slight local gains, but the inference cost increases considerably because graph-structured operations grow rapidly with the number of nodes. Therefore, placing SABE at the deeper encoder–decoder bridge stage provides a better balance between boundary representation and computational efficiency.

#### 3.3.3. Effectiveness of CDGC

CDGC is introduced into the decoder-side bridge layer to suppress background-induced spectral interference and improve discriminative feature coupling. To evaluate its effectiveness, we replace CDGC with several representative attention and feature-reweighting modules at the same position, including A^2^-Nets [31], CBAM [32], CoordAtt [33], ECA [34], an MLP-Mixer block [35], an adapted spatial–spectral attention baseline derived from the PCA-guided network of Wang et al. [36], SE [37], SimAM [38], SKNet [39], and Triplet Attention [40]. The comparison results are shown in Table 7.

As shown in Table 7, the compared attention and feature-reweighting modules show relatively close numerical results under the unified fixed-seed protocol. CDGC records the highest numerical mIoU, F1-score, Accuracy, and Specificity, reaching 68.60%, 91.07%, 86.22%, and 96.71%, respectively. CBAM records the highest numerical Sensitivity of 84.53%, while CDGC reaches 84.15%. Thus, under this fixed-seed comparison, CDGC shows a numerically favorable balance between plantation detection and background suppression.

The observed numerical pattern is consistent with the design of CDGC, which jointly models channel responses, spatial structures, and contextual interactions. This mechanism is intended to suppress false activations from bare soil, buildings, roads, and grassland, while preserving plantation canopy responses. Because repeated-seed experiments were not performed, the numerical differences among these modules are not interpreted as statistically established performance advantages.

#### 3.3.4. Effectiveness of FReSCO

To evaluate the optimization-level contribution of FReSCO, we compare Adam + FReSCO with several commonly used optimization strategies under the same model configuration. In this experiment, the network architecture and all non-optimization training settings are kept unchanged. The compared settings include Adadelta, Adagrad, Adam, Adamax, AdamW, ASGD, RMSprop, and Adam + FReSCO. The results are shown in Table 8.

As shown in Table 8, Adam + FReSCO records the highest numerical values among the compared optimization strategies under the fixed-seed protocol, reaching 67.47% mIoU, 90.55% F1-score, 85.47% Accuracy, 96.40% Specificity, and 83.32% Sensitivity. Relative to standard Adam, the observed values change from 66.25% to 67.47% for mIoU, from 90.11% to 90.55% for F1-score, from 84.80% to 85.47% for Accuracy, from 94.20% to 96.40% for Specificity, and from 82.94% to 83.32% for Sensitivity.

Under the fixed comparison protocol, Adam + FReSCO produced higher numerical values than standard Adam for all five reported metrics. FReSCO reweights the gradient using boundary-, texture-, spectral-, and scale-related terms before the Adam update; these results are consistent with an optimization-level contribution under this configuration. Because the study used one fixed seed, the differences should be interpreted only as observed numerical rankings and not as a statistically established advantage over the compared optimizer settings. FReSCO changes training only and does not add an inference-time branch.

### 3.4. Ablation Studies

To quantitatively evaluate the contribution of SIAF, SABE, CDGC, and FReSCO to the overall segmentation performance, we conduct a series of ablation experiments under the same training and evaluation settings. The results are summarized in Table 9.

Table 9 summarizes the joint ablation results of SIAF, SABE, CDGC, and FReSCO. Under the fixed-seed protocol, Group I, which integrates all four components, records the highest numerical mIoU, F1-score, Accuracy, and Sensitivity, reaching 69.36%, 91.43%, 86.72%, and 84.76%, respectively. Group H records the highest numerical Specificity of 96.71%, compared with 96.66% for the complete model. Overall, Group I records the highest numerical combination of overlap accuracy and plantation recall among the evaluated configurations, while these differences are treated as descriptive fixed-seed results.

To examine the numerical contribution of each component under a common configuration, the complete Group I configuration is compared with the corresponding three-component configurations in Groups E–H. Relative to Group E, Group I changes mIoU from 66.10% to 69.36%, F1-score from 89.93% to 91.43%, Accuracy from 84.58% to 86.72%, and Sensitivity from 82.41% to 84.76%. Relative to Group F, the numerical values change from 67.47% to 69.36% for mIoU, from 90.55% to 91.43% for F1-score, and from 85.47% to 86.72% for Accuracy. Relative to Group G, mIoU changes from 67.92% to 69.36% and F1-score from 90.77% to 91.43%. Relative to Group H, mIoU changes from 68.60% to 69.36%, F1-score from 91.07% to 91.43%, Accuracy from 86.22% to 86.72%, and Sensitivity from 84.15% to 84.76%, while Specificity changes from 96.71% to 96.66%. These are descriptive differences from the single fixed-seed runs rather than estimates of expected improvement across repeated training runs.

The two-component configurations also show that the relative effect of a module depends on the surrounding architecture. For example, Group A (SIAF + SABE) achieves 66.12% mIoU, whereas Group B (SIAF + CDGC) achieves 65.95%, indicating similar performance under these reduced configurations. Group D (SABE + CDGC) reaches 66.45% mIoU, while the addition of FReSCO in Group E results in 66.10%. Therefore, the effect of an individual component should not be interpreted as uniformly positive under every partial configuration. Instead, the results indicate that the four components are complementary when integrated within the complete KGF-Net framework.

The fixed-seed ablation results are consistent with the coordinated design of KGF-Net rather than an assumption that every module independently increases every metric in every configuration. SIAF is designed for vegetation-index-aware representation learning, SABE for structural and boundary modeling, CDGC for discriminative feature coupling under complex backgrounds, and FReSCO for optimization-level gradient modulation. The observed rankings describe this particular experimental protocol and are not presented as statistically established module-wise superiority.

**Figure 8:**
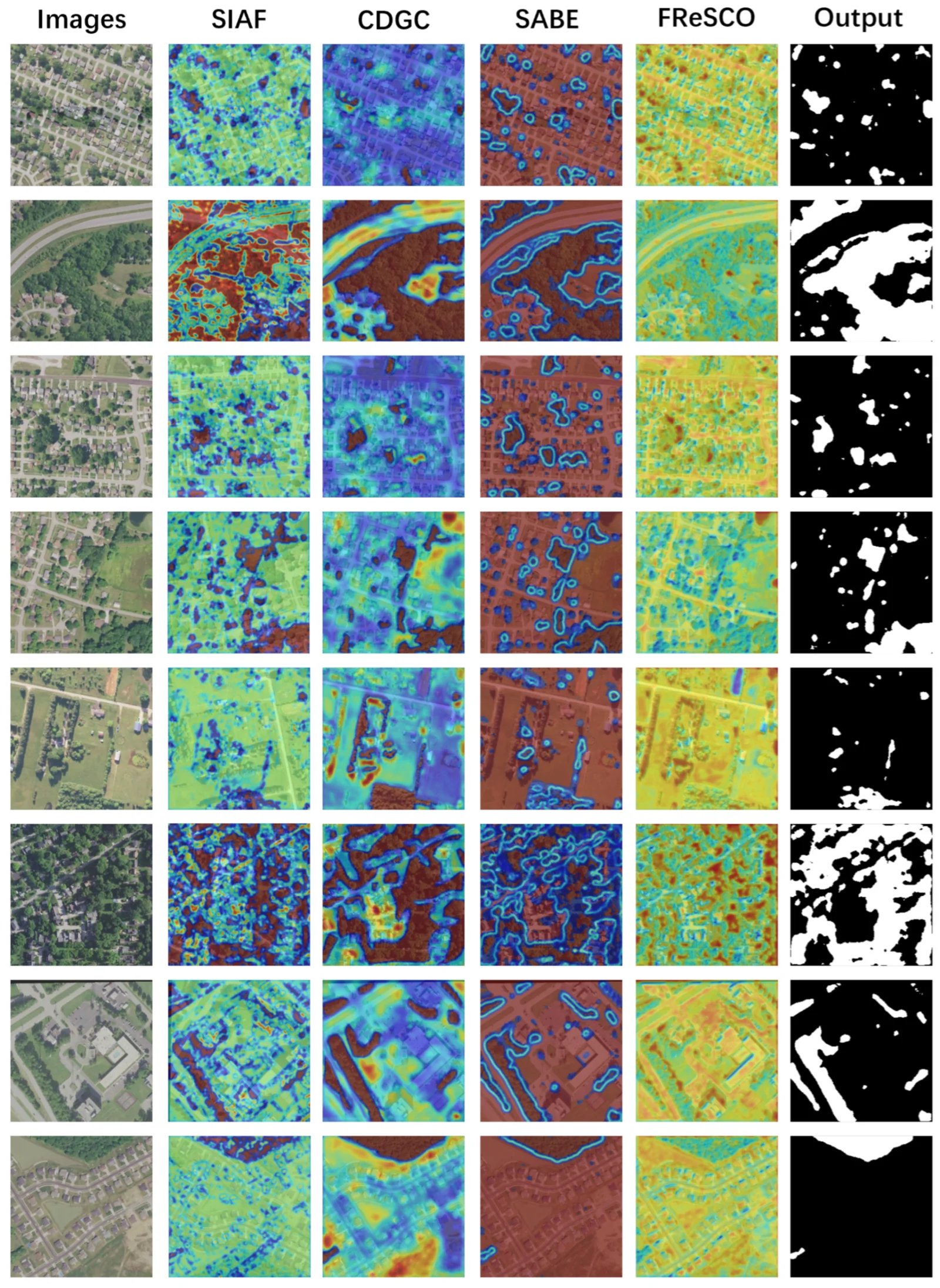
Module-wise activation maps on representative test samples. From left to right are the original image, SIAF activation map, CDGC activation map, SABE activation map, Adam + FReSCO activation map, and the corresponding ground-truth mask. Warmer colors indicate stronger model responses.

Figure 8 provides qualitative activation maps for representative test samples. In these examples, SIAF responses cover a larger fraction of the annotated plantation area, CDGC maps contain fewer strong responses over roads and built-up surfaces, and SABE responses are concentrated near visible stand contours. The Adam + FReSCO maps also appear less spatially scattered in several samples. These observations are descriptive rather than quantitative: the heatmaps illustrate behavior that is consistent with the ablation results, but they do not independently establish the contribution of each component.

### 3.5. Comparative Experiments

In this section, fourteen representative semantic segmentation models are selected as comparison baselines. These models include Transformer-based architectures, namely Swin-UNet [41] and SegFormer [42]; classical encoder–decoder structures, including UNet [43], UNet++ [44], and SegNet [45]; multi-scale context modeling networks, including PSPNet [46] and DeepLabv3 [47]; and the lightweight model BiSeNetV2 [48]. In addition, recently reported segmentation models are also included for comparison. Among them, BaAFN [13] and SegMAN [49] are used as advanced single-modality segmentation baselines, while DFormer [50], DPLNet [51], MixPrompt [52], and KTB [53] are included as multimodal baselines. This setting enables a comprehensive comparison on the North American source-domain benchmark, where KGF-Net uses the RGB–NDVI–EVI configuration and all methods follow the same scene-level data partitions and evaluation protocol.

For the North American benchmark, the conventional single-modality baselines, including UNet, UNet++, SegNet, PSPNet, DeepLabv3, BiSeNetV2, Swin-UNet, SegFormer, BaAFN, and SegMAN, were trained using RGB imagery only. The multimodal baselines, including DFormer, DPLNet, MixPrompt, and KTB, used RGB imagery as the primary modality and the spatially aligned NDVI and EVI layers as auxiliary modalities. KGF-Net used the same RGB, NDVI, and EVI information as these multimodal baselines. Therefore, KGF-Net was not provided with additional spectral information that was unavailable to the competing multimodal methods.

As shown in Table 10, KGF-Net records the highest numerical values across the five reported metrics under the fixed-seed protocol. Specifically, KGF-Net reaches 69.36% mIoU, 91.43% F1-score, 86.72% Accuracy, 96.66% Specificity, and 84.76% Sensitivity. Among the recent single-modality baselines, SegMAN records the closest mIoU, with 69.18%, while DFormer reaches 68.98% among the multimodal baselines. Because the experiments were conducted using one fixed random seed, the small 0.18-percentage-point mIoU difference between KGF-Net and SegMAN should be interpreted as an observed numerical ranking under the specified protocol, rather than evidence of statistical superiority. Therefore, the reported results demonstrate the comparative performance under the controlled evaluation setting, but they do not provide variance estimates across repeated runs. Accordingly, the comparison establishes the fixed-seed numerical position of KGF-Net on this benchmark but does not support a claim of statistically significant superiority over SegMAN or the other baselines.

Besides segmentation accuracy, practical deployment also requires careful consideration of computational cost and runtime efficiency. Therefore, KGF-Net is further compared with representative baseline models in terms of parameters, FLOPs, peak GPU memory, inference time, speed, and GPU utilization. As shown in Table 11, KGF-Net achieves the lowest number of parameters, the smallest FLOPs, and the lowest peak memory usage among all compared models, indicating a highly lightweight design. In terms of runtime efficiency, KGF-Net reaches an inference time of 8.21 ms/img and a throughput of 121.74 FPS, which is substantially faster than most mainstream multimodal, Transformer-based, and conventional CNN-based segmentation models. Although BiSeNetV2 and UNet achieve higher raw inference speed, their segmentation accuracy remains lower than that of KGF-Net, as reported in Table 10. These results indicate that KGF-Net achieves a favorable balance between segmentation accuracy and deployment efficiency.

All efficiency measurements were conducted on the same NVIDIA GeForce RTX 3090 GPU with a batch size of 1 and an input size of 500 × 500 pixels. Before formal timing, 10 forward passes were used for GPU warm-up, followed by 100 synchronized forward passes for measurement. torch.cuda.synchronize() was called before and after each timed pass. Inference time included only the network forward computation and excluded image loading, preprocessing, and post-processing. Peak GPU memory was obtained using torch.cuda.max_memory_allocated() after resetting the CUDA memory statistics. The reported inference time, FPS, peak memory, and GPU utilization were measured under this unified protocol.

**Figure 9:**
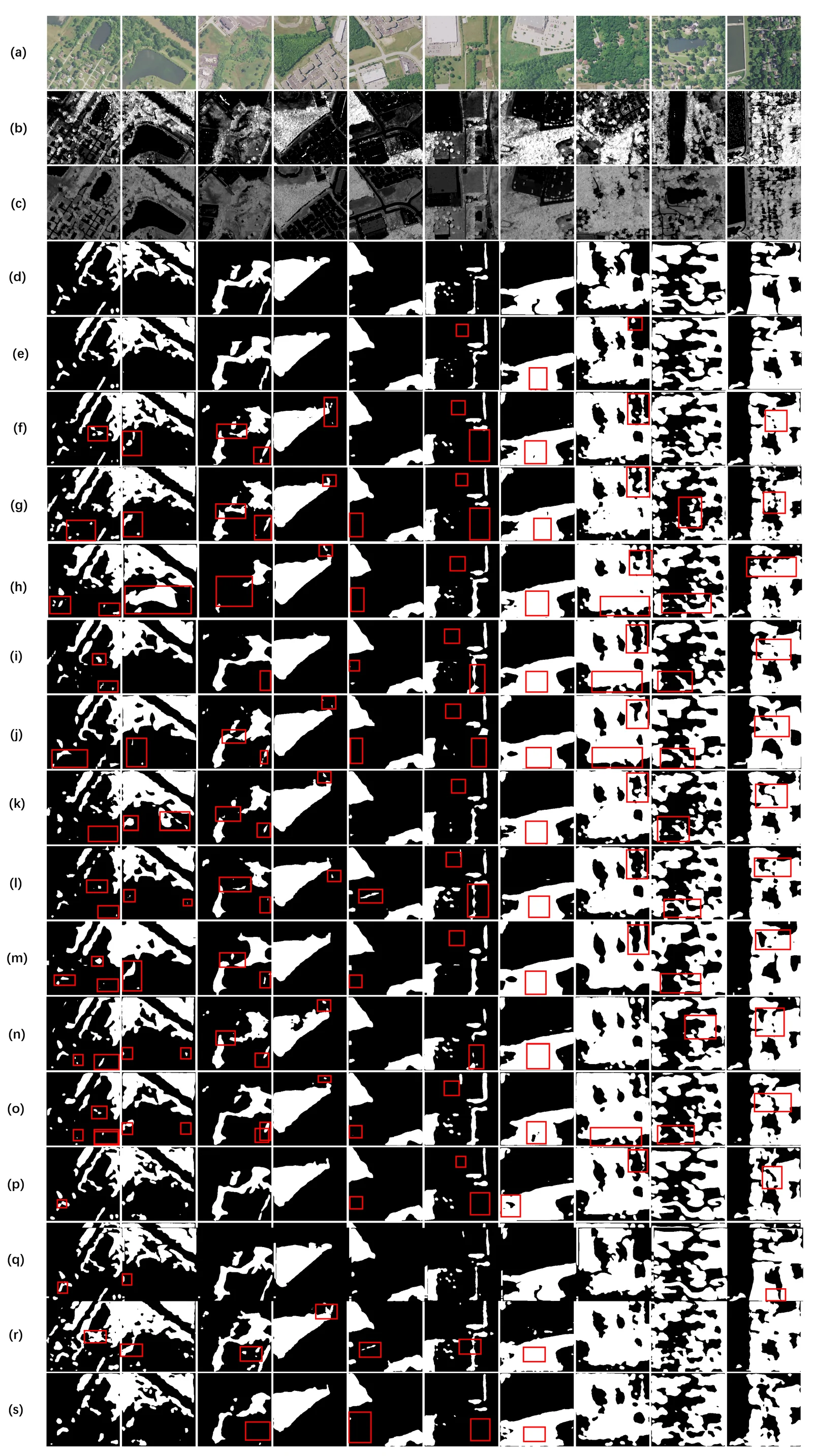
Qualitative comparison of plantation segmentation results on the benchmark dataset. (**a**) Original image, (**b**) EVI image, (**c**) NDVI image, (**d**) ground-truth mask, and (**e**–**s**) segmentation results of KGF-Net, DFormer, DPLNet, SegFormer, Swin-UNet, DeepLabv3, UNet++, UNet, BiSeNetV2, PSPNet, SegNet, BaAFN, KTB, MixPrompt, and SegMAN, respectively. Red boxes highlight representative error regions, including foreground omission, background false positives, boundary discontinuity, fragmented prediction, and over-smoothed plantation edges.

Figure 9 presents qualitative comparisons of different segmentation models on the plantation remote-sensing dataset. Red boxes indicate representative prediction errors, including boundary discontinuity, foreground omission, and background false positives. The qualitative differences among methods are discussed in detail in the following analysis.

### 3.6. Cross-Region Transfer Experiments

To evaluate source-to-target transferability, the North American subset was used as the multimodal source domain, while Asia, Europe, and Africa were treated as plantation target domains. In addition, FAIS was included as an auxiliary forest-cover benchmark to evaluate the transferability of the learned forest representation beyond plantation-specific scenarios. The source checkpoint was pretrained with North American RGB, NDVI, and EVI using an approximately 70%/10%/20% split. For each target dataset, approximately 20% of the RGB images and masks were used for fine-tuning, 10% were used for validation, and the remaining 70% were reserved exclusively for independent testing. No target-domain vegetation-index layer was generated or used, and no target test sample was used during pretraining, fine-tuning, or checkpoint selection. Accordingly, this protocol evaluates multimodal source pretraining with limited RGB-based target adaptation rather than zero-shot generalization.

For the cross-region baseline experiments, each model was first trained on the North American source-domain subset according to its supported input configuration. The conventional single-modality models used North American RGB imagery, whereas DFormer, DPLNet, MixPrompt, and KTB used RGB as the primary modality and the aligned NDVI and EVI layers as auxiliary modalities. During adaptation to Asia, Europe, Africa, and FAIS, all models were fine-tuned using the corresponding target-domain RGB images and segmentation masks only. For the multimodal architectures, the auxiliary vegetation-index pathway was disabled during target-domain fine-tuning and inference, while the source-initialized RGB pathway and shared downstream parameters were retained and updated using the target-domain RGB training data. Therefore, no baseline model or KGF-Net received target-domain vegetation-index information, and all methods were evaluated using the same target-domain RGB data and scene-level partitions.

The three self-constructed target datasets correspond to representative regions in Asia, Europe, and Africa. They contain high-resolution RGB imagery and pixel-level masks and cover variations in background complexity, illumination, terrain, and plantation structure. Their scene-level fine-tuning, validation, and test partitions followed the approximately 2:1:7 protocol described in the Data Collection subsection. The public FAIS dataset is a Kaggle-hosted RGB forest-cover segmentation dataset derived from the land-cover track of the DeepGlobe 2018 challenge [19,54]. Unlike the self-constructed datasets, FAIS does not provide plantation-specific annotations. Therefore, the FAIS experiment evaluates general forest-cover segmentation capability rather than direct plantation classification performance. It was uniformly preprocessed and divided into fine-tuning, validation, and test subsets using the same approximately 2:1:7 ratio. On each target dataset, KGF-Net and the baseline models were fine-tuned and evaluated using the same RGB target-domain partitions and metrics.

As shown in Table 12, Table 13, Table 14 and Table 15, KGF-Net records the highest numerical mIoU across all four target-domain evaluations under the specified fixed-seed transfer protocol. On the FAIS dataset, KGF-Net records the highest numerical mIoU, F1-score, Accuracy, and Specificity, while DeepLabv3 records the highest numerical Sensitivity. On the Africa subset, KGF-Net records the highest numerical mIoU, while SegMAN, DFormer, and DPLNet record the highest numerical values for individual auxiliary metrics. On the Europe subset, KGF-Net records the highest numerical mIoU, F1-score, Accuracy, and Sensitivity, whereas DFormer records the highest numerical Specificity. On the Asia subset, KGF-Net records the highest numerical mIoU, F1-score, and Accuracy, while SegFormer and DFormer record the highest numerical Specificity and Sensitivity, respectively. These rankings describe the outcomes of the specified single-seed transfer experiments and are not interpreted as evidence of statistical superiority among the compared models.

To explicitly isolate the contribution of vegetation-index-assisted source-domain pretraining, an additional controlled transfer ablation was conducted using three initialization strategies. In the first setting, KGF-Net was trained directly on each target-domain RGB subset from random initialization without North American source pretraining. In the second setting, KGF-Net was first pretrained on the North American source domain using RGB imagery only and was then adapted to each target domain using RGB imagery only. The third setting followed the proposed protocol, in which North American RGB, NDVI, and EVI were used during source-domain pretraining, followed by the same RGB-only target-domain adaptation. All three settings used identical target-domain partitions, training settings, checkpoint-selection rules, and evaluation procedures, so that the comparison isolates the effect of source-domain initialization.

As shown in Table 16, random initialization records the lowest numerical mIoU on all four target datasets, whereas RGB source pretraining records higher target-domain mIoU values under the same fixed-seed protocol. Incorporating NDVI and EVI during North American source-domain pretraining is associated with additional numerical differences of 1.99, 1.56, 2.30, and 2.23 percentage points relative to RGB-only source pretraining on Asia, Europe, Africa, and FAIS, respectively. These fixed-seed results are consistent with useful transfer from source-domain representation learning and with an additional contribution from vegetation-index-assisted source pretraining, but they are not interpreted as statistically established improvements across repeated runs.

**Figure 10:**
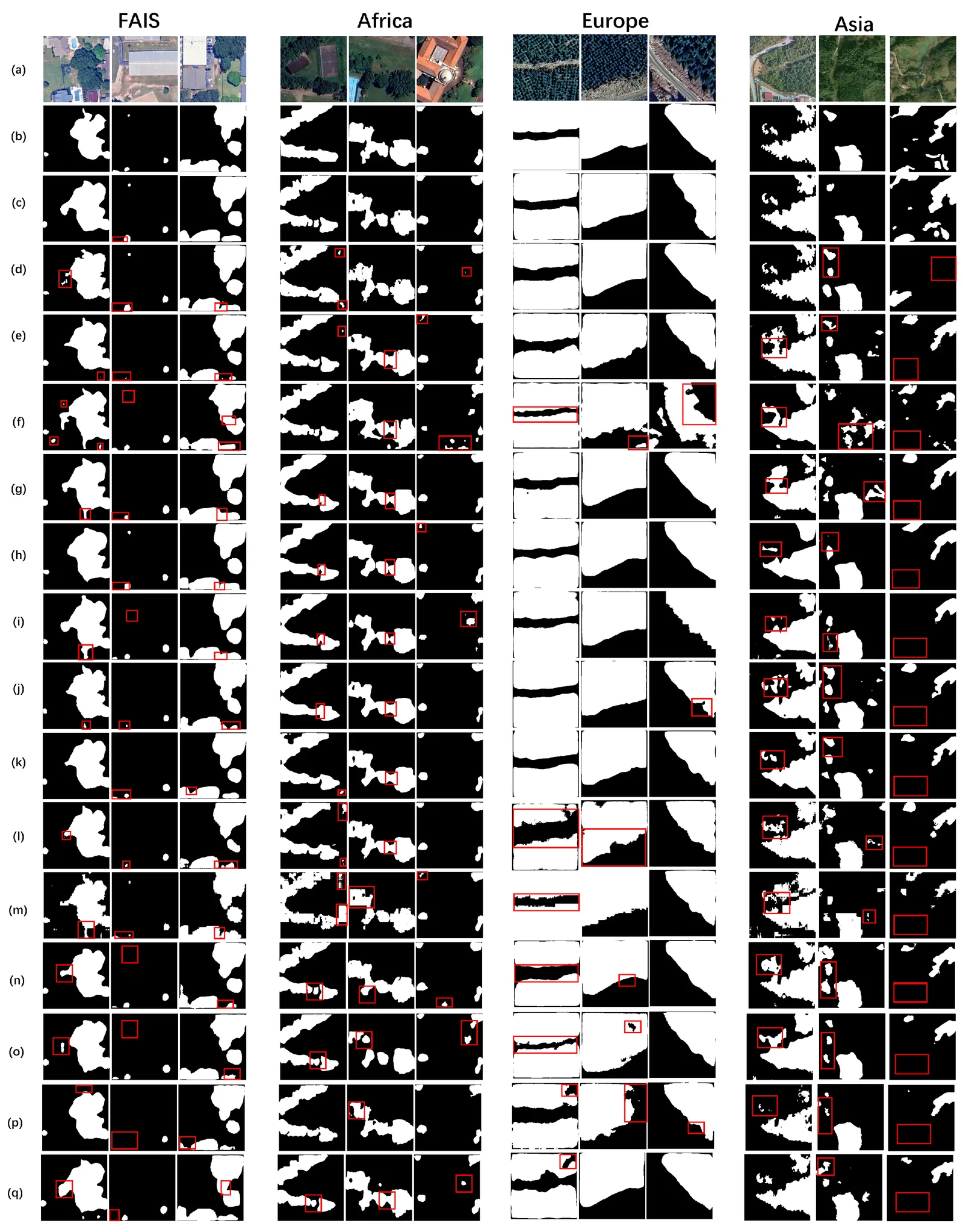
Qualitative comparison after North American RGB–NDVI–EVI pretraining and separate RGB-only target-domain fine-tuning. (**a**) Original RGB image, (**b**) ground-truth mask, and (**c**–**q**) results of KGF-Net and the fourteen baseline models. Red boxes indicate representative target-domain errors, including omission, background encroachment, fragmented boundaries, and loss of narrow plantation strips.

As shown in Figure 10, we provide visual comparisons of cross-regional samples, where regions inconsistent with the ground-truth labels, including background encroachment, foreground omission, discontinuous boundaries, and scattered false positives, are highlighted with red boxes. KGF-Net produces the fewest highlighted areas, delivering more coherent forest edges and better preservation of narrow strips and small patches. It also remains stable under strong shadows and complex textures. DFormer maintains a generally correct outline but yields softer boundaries and tends to absorb background structures near roads and buildings. DPLNet preserves the geometry of regular plantation strips relatively well but exhibits holes and small false detections in high-texture regions or near bare soil. SegFormer and Swin-UNet retain the overall shape but overly smooth fine details, causing weak-texture regions to shrink into smaller patches. DeepLabv3 benefits from strong contextual modeling but often suppresses thin branches and small objects. UNet and UNet++ show boundary jitter, breaks, and voids in noisy or rough-texture areas. BiSeNetV2 tends to miss elongated forest strips and thus shows weaker structural continuity. PSPNet and SegNet are more likely to produce large-area background spillover under strong interference. BaAFN and SegMAN show relatively competitive visual performance, but local boundary leakage and incomplete preservation of fine plantation structures can still be observed in complex scenes. MixPrompt and KTB improve cross-scene adaptability to some extent, yet fragmented predictions and false activations remain in regions with strong spectral variation.

Across the four target datasets, KGF-Net records competitive fixed-seed numerical results after North American RGB–NDVI–EVI pretraining and RGB-only target-domain fine-tuning. Together with the controlled initialization comparison in Table 16, the observed rankings are consistent with a contribution from source-domain pretraining and with additional numerical gains from vegetation-index-assisted source pretraining. These observations describe the specified single-seed protocol and do not establish statistical superiority across repeated training runs.

### 3.7. Large-Scale Plantation Mapping Results

To further evaluate the practical applicability of KGF-Net under large-scale scenarios, plantation mapping experiments were conducted in four representative study areas, including Yuan’an County, China; Stewart County, Georgia, USA; East Hampshire, UK; and Mecha, Ethiopia. The North American RGB–NDVI–EVI pretrained model was used as the source-domain initialization, followed by RGB-only fine-tuning with samples collected from each target region before large-scale inference.

**Figure 11:**
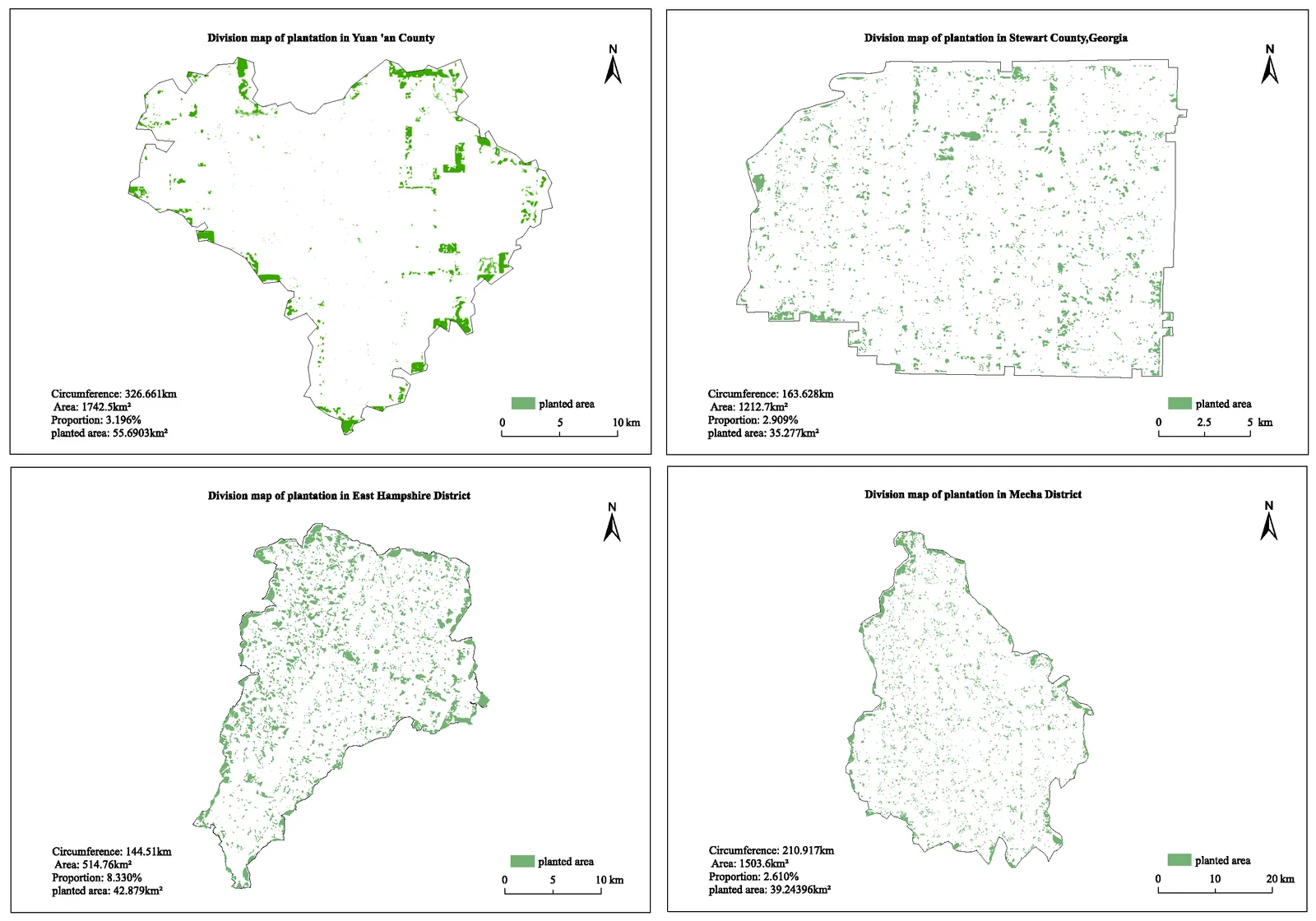
Large-scene plantation mapping results in four representative study areas: Yuan’an County, China; Stewart County, Georgia, USA; East Hampshire, UK; and Mecha, Ethiopia. Plantation regions are shown as mapped forest patches, with background areas representing non-plantation regions or reference imagery.

These study areas represent diverse plantation landscapes, including conifer plantations, conifer–broadleaf mixed plantations, plantation mosaics, and managed stands located in agro-pastoral transition regions. As illustrated in Figure 11, KGF-Net generated spatially continuous plantation segmentation maps across different geographical environments. The predicted results effectively captured plantation extent, patch distribution, and visible management boundaries, while maintaining consistent responses under variations in vegetation conditions, illumination, terrain, and surrounding land-cover complexity.

The large-scale mapping experiments further demonstrate the capability of KGF-Net to extend from benchmark-level evaluation to practical regional plantation monitoring. By integrating vegetation-index-assisted source-domain representation learning with RGB-only target-domain adaptation, the proposed framework provides a feasible solution for plantation mapping in regions where consistent multispectral observations may not be available.

## 4. Discussion

The main scientific contribution of this study is a two-stage transfer strategy that uses vegetation-related spectral information when it is available and transfers the learned representation to RGB-only regions. During North American source-domain pretraining, NDVI and EVI contribute information related to canopy greenness and vegetation condition [55], whereas RGB texture, crown arrangement, edge continuity, and scene context characterize plantation structure and management boundaries. During target-domain adaptation, Asia, Europe, Africa, and FAIS use RGB imagery only. In the controlled fixed-seed initialization experiment, RGB-only source pretraining records higher target-domain mIoU than random initialization, while RGB+NDVI+EVI source pretraining records additional numerical gains across all four target datasets. These observations are consistent with the objective of domain adaptation methods that reduce distribution discrepancies between source and target domains [56]; however, they are treated as descriptive fixed-seed results rather than evidence of statistically established superiority. However, they are treated as descriptive fixed-seed results rather than evidence of statistically established superiority.

Compared with previous plantation-mapping studies based mainly on Sentinel-2 or Landsat imagery [3,4,5,6], the present study focuses on sub-meter imagery and the explicit delineation of fine management boundaries, narrow plantation strips, and fragmented patches. Relative to high-resolution forest-characterization work based on optical imagery [7], KGF-Net additionally investigates vegetation-index-assisted source pretraining and limited RGB target adaptation across continents. The role of SABE also differs from general boundary-enhancement networks developed primarily for regular remote-sensing objects [11,12,13]: it uses hybrid static–dynamic adjacency to represent irregular plantation-edge topology. Similarly, SIAF differs from general multimodal segmentation frameworks [9,10] by using aligned vegetation indices as source-domain physiological cues and explicitly evaluating whether the learned representation remains useful when target-domain inference is restricted to RGB imagery.

The regional experiments also reveal several forestry-relevant sources of uncertainty. Young or recently established plantations may have sparse crowns and exposed soil, causing omission or confusion with shrubland and cropland. Mature closed-canopy plantations can become spectrally and texturally similar to natural forests, particularly where planting rows are no longer clearly visible. Mountain shadows, seasonal illumination differences, variable crown density, mixed-species stands, roads, and settlements further alter the appearance of plantation boundaries. Orchards and agroforestry systems remain particularly difficult because their regular planting patterns may resemble plantations, even though their land-use functions differ. These cases explain why spectral indices alone are insufficient and why boundary and contextual modeling are required in high-resolution remote-sensing segmentation studies [22].

The transfer experiments used North American RGB–NDVI–EVI pretraining followed by separate RGB-only fine-tuning with approximately 20% of each target dataset. Their results therefore demonstrate transferability after local adaptation rather than universal zero-shot applicability. This distinction is operationally important because plantation species composition, stand age, management intensity, terrain, and image characteristics vary among regions, while reliable target-domain near-infrared data may not always be available. The public FAIS dataset provides an auxiliary RGB forest-cover benchmark; because it is not plantation-specific, its results should be interpreted as evidence of transferable forest-segmentation capability rather than a direct validation of plantation classification.

The large-scale mapping experiments demonstrate the potential of KGF-Net for practical plantation monitoring applications. The generated maps provide spatial information on plantation extent, patch distribution, fragmentation, and visible management boundaries, which can support forest-resource inventory updates, afforestation-performance assessment, management-unit delineation, plantation-fragmentation analysis, and field verification prioritization. However, the binary segmentation outputs do not directly provide information on plantation species, stand age, biomass, timber volume, or carbon stock. These variables require additional field observations, temporal remote-sensing data, or dedicated retrieval models.

For the Google-basemap study areas, imagery was accessed and retrieved in October 2025 using Omap 9.8 at map level 18. This access date is distinct from the acquisition dates of the underlying satellite images. The approximate acquisition periods reported for Asia, Europe, and Africa were the temporal information available during dataset compilation, whereas exact per-scene acquisition timestamps were not consistently exposed by the basemap interface. Likewise, the spatial values reported for these subsets describe the approximate map-level-18 exported ground sampling interval calculated from the Web-Mercator ground-resolution relationship, rather than the native ground-sampling distance of an identified satellite sensor. Omap was used for study-area localization, image retrieval, scene export, and mosaicking. Because online basemap imagery may be updated, exact pixel-level reproduction may depend on the imagery version available at the time of access. Before tiling, original-scene footprints were checked for geographic overlap, and multitemporal scenes covering the same or overlapping locations were assigned to the same data subset. Large scenes were then processed using overlapping tiling, batch prediction, and weighted mosaicking, after which the outputs were georeferenced, reclassified, and vectorized in ArcMap. This workflow reduces memory requirements and supports regional mapping, although tile boundaries and changes in source imagery may introduce local uncertainty.

Several limitations remain. First, plantation labels were derived primarily from high-resolution image interpretation rather than exhaustive field inventories. Second, the current binary formulation does not distinguish plantation species, age classes, silvicultural regimes, or management objectives. Third, vegetation-index inputs were available only for the North American source domain; the target results depend on RGB fine-tuning and do not quantify the added value of target-domain indices. Fourth, cross-region performance depends on target-domain adaptation, and broader validation is required across additional ecological zones, sensors, seasons, and plantation systems. Fifth, all reported experiments used one fixed random seed. Although reporting mean values and standard deviations from multiple independent runs would provide a more complete assessment of model stability, repeated training experiments were not conducted in the current study due to computational constraints. Therefore, confidence intervals and significance tests are not available. Accordingly, all between-model and between-configuration differences should be interpreted only as observed numerical rankings under the specified protocol rather than as statistically established improvements or superiority. Sixth, BF1 was added to the controlled SABE effectiveness experiment to quantitatively assess plantation-boundary delineation, but dedicated boundary evaluation was not extended to all ablation, baseline-comparison, and cross-region experiments; complementary boundary metrics such as Boundary IoU and Hausdorff distance were also not calculated. Seventh, the patch-size statistics reported in Table 1 are calculated from connected components within individual image tiles. Because the tiling process can divide a continuous plantation stand across tile boundaries, these values should be interpreted as tile-level structural descriptors rather than direct estimates of landscape-scale plantation stand size. Finally, patch-wise inference may produce local edge artifacts, while online basemap updates may affect exact image reproducibility.

## 5. Conclusions

This study addresses fine-grained plantation mapping through multimodal source pretraining and RGB-based target adaptation. KGF-Net integrates SIAF, SABE, CDGC, and FReSCO to learn vegetation-sensitive spectral and structural representations from North American RGB, NDVI, and EVI data and to transfer the shared segmentation parameters to RGB-only target regions.

On the North American benchmark, KGF-Net records 69.36% mIoU and 91.43% F1-score. In the cross-region experiments, North American RGB–NDVI–EVI pretraining followed by RGB-only fine-tuning on approximately 20% of each target dataset records the highest numerical mIoU on the three plantation regions (Africa, Europe, and Asia) and the independent FAIS forest-cover benchmark under the specified fixed-seed protocol. The controlled transfer ablation also records higher numerical mIoU after RGB-only source pretraining than after random initialization, with additional numerical gains for RGB+NDVI+EVI source pretraining. These results are consistent with vegetation-index-assisted source-domain representation learning as a useful initialization strategy for RGB-only cross-region adaptation, but they are not presented as statistically established superiority because repeated-seed experiments were not conducted. The 1.28 M parameters, 1.64 G FLOPs, 0.89 GB peak memory, and 121.74 FPS further indicate practical deployment potential.

From a forestry-application perspective, the resulting plantation maps can support forest-resource inventory updates, afforestation-performance assessment, management-unit delineation, plantation monitoring, and field-survey prioritization. The outputs should nevertheless be interpreted as plantation-boundary products rather than direct estimates of species, stand age, biomass, timber volume, or carbon stock. Future work will evaluate target-domain multispectral inputs where available and compare RGB-only adaptation with full multimodal target fine-tuning.

## Figures and Tables

**Table 1:** Dataset-level class composition and spatial-structure statistics derived from ground-truth masks. Patch-size statistics represent connected plantation components within individual image tiles. FAIS is excluded from plantation-specific statistics because it provides forest cover rather than plantation annotations.

Region	Plantation Pixel Ratio (%)	Background Pixel Ratio (%)	Mean Patch Size (Pixels)	Median Patch Size (Pixels)	Boundary Pixel Ratio (%)
North America	83.54	16.46	18452.6	12630	18.72
Asia	52.36	47.64	8421.3	5368	27.84
Europe	51.20	48.80	7654.8	4821	29.15
Africa	51.27	48.73	9135.7	6042	26.93

**Table 2:** Methodological novelty and plantation-specific contribution of the four KGF-Net components.

Component	Limitation Addressed	Methodological Novelty Relative to Conventional Designs	Plantation-Specific Contribution
SIAF	RGB-only models and simple concatenation do not explicitly align vegetation-sensitive and spatial representations.	Performs pixel-level, same-scale alignment of RGB features with NDVI/EVI features through VI-FEM, cross-modal fusion, dual attention, and feature correction during source-domain pretraining; the learned, shared representation is then transferred to RGB-only target adaptation.	Improves discrimination between plantations and visually similar natural vegetation by jointly encoding canopy texture, greenness, density, and vegetation condition.
SABE	Convolutional boundary modules and generic graph operators inadequately preserve fragmented, irregular, or narrow plantation edges.	Constructs a hybrid relational graph that combines static grid adjacency and dynamically learned semantic adjacency, regulated by distance decay, elastic edge-weight shaping, and adaptive gating.	Strengthens the continuity and topology of stand boundaries, shelterbelts, narrow strips, and fragmented plantation patches under canopy overlap and texture perturbation.
CDGC	Single channel- or spatial-attention mechanisms can amplify roads, buildings, bare soil, cropland, and other plantation-like backgrounds.	Jointly couples channel recalibration, multi-scale spatial attention, interaction gating, and global context encoding within a residual feature-fusion structure.	Suppresses false plantation responses from heterogeneous backgrounds while retaining canopy responses and fine structural details across different plantation scales.
FReSCO	Standard optimizers apply relatively uniform gradient updates and do not explicitly account for plantation boundaries, textures, spectral imbalance, or scale variation.	Modulates the back-propagated gradient using boundary-, texture-, spectral-, and scale-aware factors before the Adam update; it is an optimization-layer strategy rather than an independent optimizer and adds no inference-time branch.	Stabilizes training under cross-region spectral variation, emphasizes difficult boundary regions, and improves transfer robustness without increasing inference complexity.

**Table 3:** Experimental hardware and software environment.

Category	Item	Configuration Item	Version
Hardware	CPU	AMD Ryzen 7 5800H	–
Hardware	RAM	64 GB	–
Hardware	GPU	NVIDIA GeForce RTX 3090	–
Hardware	Video memory	24 GB	–
Software	Operating system	Linux	–
Software	CUDA	–	11.7
Software	cuDNN	–	8.2.1
Software	PyTorch	–	1.10.1
Software	Torchvision	–	0.11.2
Software	Python	–	3.8
Software	timm	–	0.4.12
Software	scipy	–	1.2.1
Software	h5py	–	3.6.0
Software	mamba_ssm	–	1.0.1
Software	matplotlib	–	3.5.1

**Table 4:** Main training and optimization settings used for KGF-Net.

Setting	Value
Input size	500 × 500 pixels
Source-domain input	3 RGB + 2 vegetation-index channels (NDVI and EVI)
Target-domain input	3 RGB channels only
Source-domain split	Approximately 70% training, 10% validation, and 20% test
Target-domain split	Approximately 20% fine-tuning, 10% validation, and 70% test
Training batch size	8
Validation/test batch size	1
Training epochs	300
Loss function	BCE loss + Dice loss
Loss weights	$w_{\mathrm{BCE}}=1$, $w_{\mathrm{Dice}}=1$
Optimization strategy	Adam + FReSCO gradient modulation
Initial learning rate	1 × 10^−3^
Adam coefficients	$\beta_{1}=0.9$, $\beta_{2}=0.999$
Weight decay	1 × 10^−2^
Learning-rate scheduler	CosineAnnealingLR
Scheduler parameters	$T_{\max}=50$, $\eta_{\min}=1\times 10^{-5}$
Random seed	42
Mixed precision	Disabled
Checkpoint criterion	Minimum validation loss
Target-domain protocol	Source initialization + limited RGB fine-tuning
Prediction threshold	0.5
Early stopping	Not used

**Table 5:** Effectiveness evaluation of the SIAF fusion module. The highest numerical value in each metric column under the fixed-seed protocol is shown in bold and underlined.

Fusion Strategy	Input	mIoU (%)	F1-Score (%)	Accuracy (%)	Specificity (%)	Sensitivity (%)
None	VIS	63.88	89.47	83.68	87.29	82.97
Channel Concat	VIS+GNDVI	64.91	89.87	84.29	88.84	83.40
	VIS+SAVI	64.73	89.80	84.19	88.56	83.33
	VIS+EVI	65.71	90.18	84.76	90.06	83.72
	VIS+NDVI	65.59	90.14	84.70	89.73	83.71
	VIS+NDVI+EVI	66.59	90.53	85.29	91.12	84.14
FiLM	VIS+GNDVI	66.00	90.42	85.07	88.86	84.32
	VIS+SAVI	65.33	90.10	84.61	88.69	83.81
	VIS+EVI	66.76	90.70	85.50	90.02	84.61
	VIS+NDVI	66.59	90.58	85.35	90.40	84.36
	VIS+NDVI+EVI	67.72	91.08	86.08	90.97	**85.11**
Dual-stream	VIS+GNDVI	66.37	90.51	85.23	90.03	84.28
	VIS+SAVI	66.51	90.57	85.32	90.11	84.38
	VIS+EVI	67.47	90.94	85.89	91.22	84.84
	VIS+NDVI	67.44	90.91	85.85	91.45	84.74
	VIS+NDVI+EVI	68.14	91.16	86.23	92.50	85.00
SIAF	VIS+GNDVI	67.16	90.56	85.43	94.24	83.69
	VIS+SAVI	67.02	90.44	85.28	94.93	83.38
	VIS+EVI	68.55	91.14	86.28	95.39	84.49
	VIS+NDVI	68.71	91.22	86.39	95.41	84.61
	VIS+NDVI+EVI	**69.36**	**91.43**	**86.72**	**96.66**	84.76

**Table 6:** Comparison of SABE with representative graph convolution modules. BF1 denotes the Boundary F1-score calculated with a 2-pixel matching tolerance. The highest numerical value in each metric column under the fixed-seed protocol is shown in bold and underlined.

Method	mIoU (%)	F1-Score (%)	Accuracy (%)	Specificity (%)	Sensitivity (%)	BF1 (%)
GCN	67.15	90.60	85.46	93.76	83.83	82.11
APPNP	67.38	90.66	85.57	94.39	83.83	82.38
ChebConv	67.36	90.70	85.60	93.77	**84.01**	82.46
GATConv	67.05	90.38	85.22	95.93	83.11	81.74
Gated GraphConv	66.83	90.41	85.21	94.07	83.47	81.52
GINConv	66.52	90.31	85.05	93.48	83.38	81.21
SAGEConv	67.55	90.58	85.52	**96.51**	83.35	82.72
TAGConv	67.32	90.58	85.48	95.03	83.60	82.31
SABE	**67.92**	**90.77**	**85.78**	96.46	83.67	**83.64**

**Table 7:** Comparison of CDGC with representative attention and feature-reweighting designs. Values are reported in percentage, with the best results highlighted in bold and underlined.

Method	mIoU (%)	F1-Score (%)	Accuracy (%)	Specificity (%)	Sensitivity (%)
A^2^-Nets	67.48	90.66	85.59	94.98	83.74
CBAM	68.12	91.04	86.10	94.07	**84.53**
CoordAtt	67.39	90.64	85.55	94.74	83.74
ECA	66.51	90.13	84.87	95.63	82.74
MLP-Mixer	66.57	90.12	84.86	96.20	82.63
PCA	66.93	90.35	85.16	95.59	83.11
SE	67.47	90.67	85.60	94.77	83.79
SimAM	67.02	90.39	85.23	95.59	83.18
SKNet	66.14	89.96	84.62	95.46	82.48
TripAtt	67.48	90.66	85.59	95.04	83.72
CDGC	**68.60**	**91.07**	**86.22**	**96.71**	84.15

**Table 8:** Comparison of Adam + FReSCO with commonly used optimization strategies. Values are reported in percentages. The highest numerical value in each metric column under the fixed-seed protocol is shown in bold and underlined.

Optimization Strategy	mIoU (%)	F1-Score (%)	Accuracy (%)	Specificity (%)	Sensitivity (%)
Adadelta	64.60	89.37	83.72	92.80	81.93
Adagrad	66.30	90.07	84.76	95.10	82.72
Adam	66.25	90.11	84.80	94.20	82.94
Adamax	66.95	90.38	85.20	95.30	83.21
AdamW	66.90	90.34	85.15	95.45	83.12
ASGD	64.85	89.47	83.87	93.20	82.03
RMSprop	67.20	90.47	85.34	95.75	83.29
Adam + FReSCO	**67.47**	**90.55**	**85.47**	**96.40**	**83.32**

**Table 9:** Ablation study of SIAF, SABE, CDGC, and FReSCO. Metric values are reported in percentage. The highest numerical value in each metric column under the fixed-seed protocol is shown in bold and underlined. “√” indicates that the corresponding component is included, whereas “–” indicates that it is excluded.

Group	SIAF	SABE	CDGC	FReSCO	mIoU (%)	F1-Score (%)	Accuracy (%)	Specificity (%)	Sensitivity (%)
A	√	√	–	–	66.12	90.07	84.72	93.98	82.90
B	√	–	√	–	65.95	89.92	84.54	94.70	82.54
C	√	–	–	√	66.20	89.96	84.63	95.78	82.44
D	–	√	√	–	66.45	90.14	84.86	95.16	82.83
E	–	√	√	√	66.10	89.93	84.58	95.59	82.41
F	√	–	√	√	67.47	90.55	85.47	96.40	83.32
G	√	√	–	√	67.92	90.77	85.78	96.46	83.67
H	√	√	√	–	68.60	91.07	86.22	**96.71**	84.15
I	√	√	√	√	**69.36**	**91.43**	**86.72**	96.66	**84.76**

**Table 10:** Comparison of the performance of KGF-Net and other segmentation models. Metric values are reported in percentages. The highest numerical value in each metric column under the fixed-seed protocol is shown in bold and underlined.

Network	mIoU (%)	F1-Score (%)	Accuracy (%)	Specificity (%)	Sensitivity (%)
KGF-Net	**69.36**	**91.43**	**86.72**	**96.66**	**84.76**
SegMAN	69.18	91.37	86.63	96.30	84.73
DFormer	68.98	91.30	86.52	96.00	84.65
MixPrompt	68.36	91.04	86.14	95.60	84.28
DPLNet	67.89	90.82	85.83	95.50	83.93
BaAFN	67.82	90.77	85.77	95.70	83.81
KTB	67.24	90.47	85.35	96.00	83.25
SegFormer	66.75	90.32	85.10	94.80	83.19
Swin-UNet	66.63	90.28	85.04	94.50	83.18
DeepLabv3	65.68	89.84	84.41	94.00	82.52
PSPNet	65.54	89.78	84.32	93.80	82.46
UNet++	64.95	89.50	83.92	93.50	82.03
BiSeNetV2	64.79	89.37	83.76	94.00	81.74
UNet	63.54	88.65	82.78	94.50	80.48
SegNet	62.42	88.19	82.10	92.50	80.05

**Table 11:** Efficiency comparison of KGF-Net and other segmentation models under the same batch-size-1.

Network	Params (M)	FLOPs (G)	Peak Memory (GB)	Inference Time (ms/img)	Speed (FPS)	GPU Utilization
KGF-Net	1.28	1.64	0.89	8.21	121.74	46.8
DFormer	26.44	21.78	3.19	21.65	46.19	69.5
DPLNet	16.28	14.83	2.95	17.09	58.51	61.7
SegFormer	3.75	5.85	1.96	10.24	97.67	48.9
Swin-UNet	27.85	18.63	3.52	19.75	50.64	67.2
DeepLabv3	39.63	31.45	3.28	18.02	55.49	71.4
PSPNet	46.21	34.11	3.42	19.38	51.59	72.6
UNet++	4.29	4.91	2.47	9.21	108.53	45.3
BiSeNetV2	3.41	4.29	1.59	6.18	161.71	39.8
UNet	2.68	2.42	2.21	7.35	136.12	37.6
SegNet	29.45	28.74	2.98	18.77	53.29	68.8
SegMAN	2.87	2.96	2.39	7.98	125.27	44.7
BaAFN	6.84	6.76	2.70	7.89	126.75	47.1
KTB	94.41	21.64	3.28	76.80	13.02	74.9
MixPrompt	84.42	17.52	1.72	50.88	19.66	56.3

**Table 12:** Comparative performance on the FAIS dataset. Metric values are reported in percentage, with the best results highlighted in bold and underlined.

Rank	Network	mIoU (%)	F1-Score (%)	Accuracy (%)	Specificity (%)	Sensitivity (%)
1	KGF-Net	**85.14**	**91.78**	**91.98**	**95.52**	88.52
2	SegMAN	84.68	91.56	91.71	94.58	88.90
3	DFormer	84.39	91.33	91.54	95.12	88.04
4	MixPrompt	84.37	91.47	91.52	93.22	89.87
5	DPLNet	84.35	91.57	91.51	91.93	91.10
6	BaAFN	83.41	90.95	90.95	92.10	89.84
7	KTB	83.30	90.90	90.89	91.88	89.92
8	SegFormer	83.24	90.87	90.85	91.71	90.02
9	DeepLabv3	82.61	90.65	90.48	89.67	**91.27**
10	Swin-UNet	82.41	90.52	90.36	89.69	91.01
11	UNet++	82.40	90.46	90.35	90.31	90.39
12	BiSeNetV2	81.81	90.20	90.00	89.02	90.95
13	UNet	79.34	88.69	88.48	87.73	89.22
14	PSPNet	78.44	88.18	87.92	86.79	89.03
15	SegNet	77.73	87.59	87.47	87.52	87.42

**Table 13:** Comparative performance on the Africa subset. Metric values are reported in percentage, with the best results highlighted in bold and underlined.

Rank	Network	mIoU (%)	F1-Score (%)	Accuracy (%)	Specificity (%)	Sensitivity (%)
1	KGF-Net	**85.65**	91.99	92.28	93.35	91.15
2	SegMAN	85.63	**92.26**	92.26	93.68	90.88
3	DFormer	85.56	91.27	**92.32**	92.97	**91.49**
4	DPLNet	84.65	91.69	91.69	**94.53**	89.01
5	SegFormer	83.47	90.99	90.99	93.98	88.19
6	Swin-UNet	83.10	90.77	90.77	91.49	90.06
7	MixPrompt	82.52	90.42	90.42	92.32	88.60
8	DeepLabv3	81.70	89.93	89.93	92.36	87.62
9	UNet++	79.73	88.72	88.72	89.37	88.08
10	BiSeNetV2	79.16	88.37	88.37	89.69	87.09
11	UNet	77.31	87.20	87.20	90.03	84.55
12	BaAFN	76.42	86.74	86.63	89.97	83.59
13	KTB	75.86	86.18	86.27	89.36	83.35
14	PSPNet	75.32	85.92	85.92	89.35	82.75
15	SegNet	75.25	85.88	85.88	90.61	81.62

**Table 14:** Comparative performance on the Europe subset. Metric values are reported in percentage, with the best results highlighted in bold and underlined.

Rank	Network	mIoU (%)	F1-Score (%)	Accuracy (%)	Specificity (%)	Sensitivity (%)
1	KGF-Net	**82.86**	**91.53**	**90.71**	91.11	**90.39**
2	SegMAN	82.34	91.12	90.38	90.99	89.88
3	DFormer	81.40	90.70	89.83	**91.53**	88.49
4	MixPrompt	80.92	90.28	89.51	91.01	88.29
5	DPLNet	80.40	90.95	89.42	88.92	89.76
6	BaAFN	79.56	89.34	88.66	89.39	88.04
7	KTB	79.18	88.97	88.41	89.47	87.48
8	SegFormer	78.97	88.25	88.25	89.33	87.19
9	Swin-UNet	77.51	87.33	87.33	89.47	85.29
10	DeepLabv3	76.13	86.45	86.45	85.13	87.80
11	BiSeNetV2	74.35	85.29	85.29	83.58	87.07
12	UNet++	74.25	85.22	85.22	85.78	84.67
13	UNet	71.86	83.63	83.63	84.66	82.62
14	PSPNet	71.57	83.43	83.43	84.62	82.28
15	SegNet	70.52	75.40	85.00	90.73	72.64

**Table 15:** Comparative performance on the Asia subset. Metric values are reported in percentage, with the best results highlighted in bold and underlined.

Rank	Network	mIoU (%)	F1-Score (%)	Accuracy (%)	Specificity (%)	Sensitivity (%)
1	KGF-Net	**75.98**	**86.35**	**86.35**	89.25	83.63
2	SegMAN	75.61	86.21	86.11	89.18	83.29
3	DFormer	74.43	85.34	85.34	86.58	**84.13**
4	MixPrompt	73.28	84.76	84.58	87.62	81.82
5	BaAFN	72.46	84.28	84.03	88.06	80.49
6	DPLNet	72.37	83.97	83.97	86.82	81.30
7	SegFormer	72.06	85.09	83.86	**90.96**	78.81
8	Swin-UNet	71.80	83.58	83.59	87.25	80.22
9	KTB	71.38	83.41	83.30	87.10	79.87
10	DeepLabv3	70.64	82.79	82.79	88.68	77.64
11	UNet++	70.63	82.79	82.79	85.76	80.01
12	BiSeNetV2	69.70	82.15	82.14	85.37	79.16
13	UNet	69.65	82.11	82.11	86.11	78.47
14	PSPNet	66.69	80.02	80.02	84.32	76.14
15	SegNet	66.09	79.58	79.58	83.88	75.71

**Table 16:** Effect of source-domain pretraining strategy on RGB-only target-domain adaptation. Only mIoU is reported for this controlled transfer comparison. The highest numerical value in each column under the fixed-seed protocol is shown in bold and underlined.

Initialization Strategy	Asia mIoU (%)	Europe mIoU (%)	Africa mIoU (%)	FAIS mIoU (%)
Random initialization	70.32	78.23	79.87	79.70
RGB source pretraining	73.99	81.30	83.35	82.91
RGB+NDVI+EVI source pretraining	**75.98**	**82.86**	**85.65**	**85.14**

## Data Availability

The public dataset repository is available at https://huggingface.co/datasets/jkhsdfja/ArtificialForest-HRSegDataset (accessed on 3 August 2026) and is identified as Version 1.0.0. The repository includes a dataset card, metadata description, exact split files, and a mixed-rights license notice. Author-created annotations, masks, metadata, and processing descriptions are released under the stated dataset terms, whereas third-party imagery remains subject to the original provider’s conditions and is not relicensed or redistributed where those conditions restrict redistribution. The North American source-domain subset includes the released NAIP-derived RGB images, pixel-level masks, and available NDVI and EVI layers. The Asia, Europe, and Africa target subsets contain RGB images and masks only; no target-domain vegetation-index layers were used in the reported transfer experiments. The source code is publicly available at https://github.com/LT-12345/KGF-Net.git (accessed on 3 August 2026) under the MIT License and includes the model implementation, data-processing procedures, configuration files, training and evaluation scripts, and table/figure-generation utilities. data-processing procedures, configuration files, training and evaluation scripts, and table/figure-generation utilities. Trained weights are not separately released; however, the versioned data, exact split files, code, configurations, and complete training protocol are sufficient to regenerate the reported training-based experiments, tables, and figures.
